# Adhesion chemistry of plant-based cellulosic fibers and polymeric matrices in composites: a review

**DOI:** 10.1039/d5ra09241h

**Published:** 2026-01-29

**Authors:** Md Shadhin, Nusrat Jahan Tisha, Marzia Dulal, Raghavan Jayaraman, Mashiur Rahman

**Affiliations:** a Department of Biosystems Engineering, University of Manitoba R3T 2N2 Canada Mashiur.Rahman@umanitoba.ca; b Composite Materials and Structures Research Group, Department of Mechanical Engineering, University of Manitoba Winnipeg MB R3T 5V6 Canada; c Department of Textile Engineering Management, Bangladesh University of Textiles Dhaka-1208 Bangladesh

## Abstract

Plant-based cellulosic fibers are often considered a preferred material choice for polymer composites due to growing environmental concerns. The hierarchical macro- and micro-structures of the reinforcing fibers determine the characteristics of fiber–matrix interfaces and performance of the composite. The non-aligned spatial orientation of cellulosic fibers (when used as discontinuous fibers in composites), variability in the fiber length and diameter, and heterogeneity in the chemical composition exert complex effects on the adhesion behavior in composites. Additionally, cellulosic fiber polymer composites exhibit limited interfacial compatibility with polymer matrices due to the hydrophilicity of the reinforcing materials. The review herein presents the dominant adhesion mechanisms of mechanical interlocking, chemical bonding, physical adhesion, and weak boundary layers and their impact on composite properties. Furthermore, this review studies the relationship between the cellulosic fiber structure–morphology–topography and adhesion mechanisms to address and counteract adhesion problems. For a given fiber diameter, an increase in the fiber length (up to a threshold value) increases the composite properties due to enhancement in adhesion properties. An increase in the fiber length enhances the mechanical interlocking within composites and is beneficial until it induces a curling effect. However, interfacial adhesion in composites decreases with the increase in the fiber or yarn twist due to the increase in compactness, decrease in porosity, and possible reorientation of the twisted fibers or yarns from the loading direction. The percentage cellulose content primarily determines the interface properties, while the non-cellulosic components, such as hemicellulose and lignin, contribute to the formation of weak boundary layers, adversely affecting the fiber–matrix adhesion behavior.

## Abstract 2

The adhesion chemistry of plant-based cellulosic fiber polymer composites was studied, while elucidating the composite manufacturing process, green chemistry of cellulosic reinforcement, and factors affecting composite performance.

## Graphical abstract

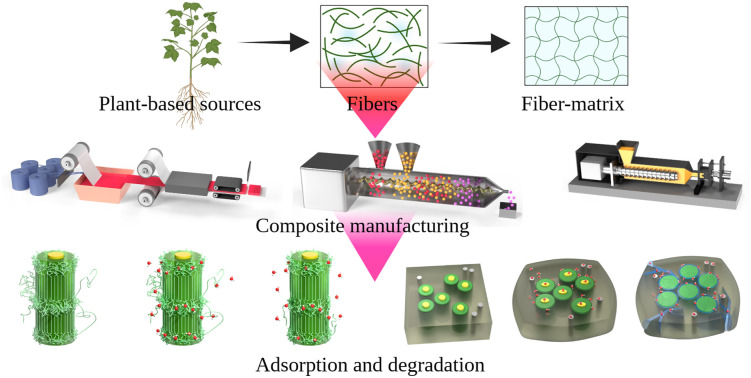

## 1. Introduction

The consistently growing interest in environmental sustainability and biobased products has led to the increased utilization of natural fibers as viable alternatives to synthetic fibers in engineering composites. Biobased renewable fibers are derived from plant and animal sources and have gained significant attention due to their lightweight nature, renewability, biodegradability, and favorable mechanical properties.^1,2^ Most of the plant-based cellulosic fibers, *i.e.*, jute, sisal, hemp, coir, flax, and pineapple leaf fibers, are particularly interesting because of their hierarchical cellulosic structures and comparable mechanical properties, while animal fibers, like wool and silk, offer additional functionalities because of the presence of proteins in their structure.^3,4^ These fibers have excellent sustainability benefits because they reduce energy consumption during processing and serve as CO_2_-neutral alternatives to their synthetic counterparts. Compared with traditional fossil-based materials, such as glass fiber (density: ∼2.5 g cm^−3^), cellulosic fibers are a lighter yet structurally strong alternative, making them attractive candidates for sustainable and eco-friendly bioproducts, such as films, composites, and nanocomposites, in various applications.^5–8^

Unlike engineering materials, like metals and steels, cellulosic fibers offer greater flexibility in design and manufacturing to tailor their properties based on end-use applications. Cellulosic fibers contribute to carbon sequestration, further reinforcing their environmental advantages.^9–11^ Over the past decade, researchers have extensively investigated emerging lignocellulosic fibers, such as canola, cattail, and newly discovered high-cellulosic fibers from *Elettaria cardamomum* (cardamom), *Epipremnum aureum* (golden pothos), and *Arundo donax*, demonstrating their feasibility in polymer composite materials.^12–14^

The macro- and micro-structures of the reinforcing fibers, such as fiber chemical composition, fiber length, diameter, aspect ratio, and linear density, determine the characteristics of the fiber–matrix interfaces and composite performance. Petroleum-based fibers, such as carbon and glass, are often manufactured using melt and wet-spinning and utilized as continuous oriented composites. Dry spinning the cellulosic fibers into continuous yarns is expensive. Hence, they are often used as discontinuous fibers. These discontinuous fibers typically exhibit a non-aligned spatial orientation in the composites. Furthermore, regulating the fiber length, diameter, and density in discontinuous fiber composites is a major challenge due to the inherent variability of plant-based materials, which to date remains beyond control. These variations, together with the large scatter in the cellulosic fiber composition and complexities in polymeric structures, cause complex local shear and make it difficult to predict the adhesion behavior and model the composite properties.

Furthermore, cellulosic fiber polymer composites exhibit susceptibility to degradation and limited interfacial compatibility with polymer matrices due to the hydrophilicity of the reinforcing materials. This is because the hydrophilic reinforcing materials exhibit poor interfacial adhesion when they are combined with hydrophobic polymer matrices, negatively impacting the mechanical performance.^15^ Also, these composites adsorb and interact with water molecules at the micro- and nano-scales, causing degradation. To address these challenges, researchers utilized coupling agents and additives, and employed different surface modification techniques to enhance the compatibility, fiber–matrix adhesion, interfacial shear strength, and durability of cellulosic fiber composites.^16–21^ Bio-based coupling agents, such as enzymatically treated lignin, tannins, and plant silanes, are a greener option than traditional synthetic treatment. The agents introduce functional groups (*e.g.*, carboxyl, hydroxyl, or amine) that enhance interfacial adhesion with the polymer matrix and fibre. Laccase or peroxidase enzymatic treatments, for instance, can oxidize lignin moieties selectively, enhancing compatibility without compromising the fiber structure.^22^ However, employing surface treatment alone is insufficient to understand the adhesion chemistry and mechanism, and optimize the composite performance. The hierarchical polymeric structure of cellulosic fiber determines their physical, chemical, topographical, and mechanical properties. These properties exhibit complex variability and critically influence the resulting interfacial bonding within the composite structure. However, to date, limited knowledge is available on complex structure–property–adhesion relationships, particularly with respect to the underlying fundamental adhesion chemistry. Hence, this review aims to bridge that gap by comprehensively analyzing the green chemistry of plant-based cellulosic fibers, adhesion theories, surface modification techniques, interfacial bonding strategies, and the relationship between the cellulosic fiber structure–morphology–topography and adhesion mechanisms to address and counteract the adhesion problems, ultimately contributing to the advancement of next-generation sustainable composite materials.

## 2. Cellulosic fibers

### 2.1 Structure, composition, and applications

**Fig. 1:**
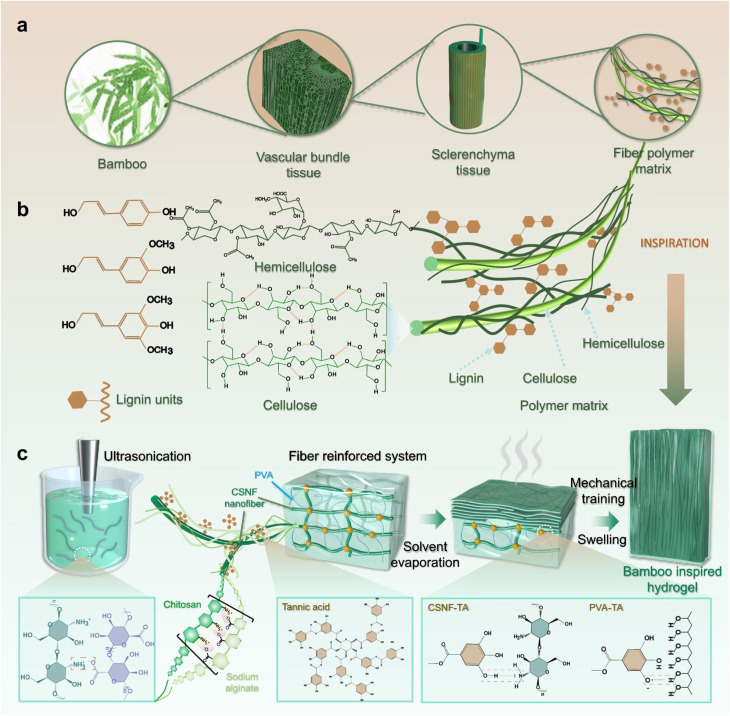
Multiscale representation of the hierarchical structure of cellulosic plant fibers and applications. (a) Hierarchical structure of cellulosic fibers (bamboo). (b) Fundamental cellulose–lignin–hemicellulose composition unit of bamboo. (c) Applications of cellulosic fibers in composite materials. This figure has been adapted from ref. 23 with unrestricted permission from Springer Nature, licensed under CC BY-NC-ND 4.0 International License, copyright © 2025.

Plant-based cellulosic fiber features a hierarchical structure fundamentally composed of cellulose, hemicellulose, lignin, and pectin. The hierarchical structure of a cellulosic fiber is shown schematically in Fig. 1a and b, emphasizing the complexity of its fibril layers. These fibers hold a fiber-reinforced polymer composite structure, where cellulose microfibrils act as the reinforcing elements embedded in a lignin and pectin polymer matrix. Cellulose microfibrils, being crystalline and serving as the primary reinforcing elements within the fiber-reinforced polymer composite structure, largely determine the mechanical strength, stiffness, and overall structural properties of the plant-based cellulosic fiber. Hemicellulose acts as a cohesive matrix that holds cellulose and lignin together and determines the flexibility. In contrast, lignin and pectin fill the voids between the cellulose fibrils, providing stiffness and resistance to deformation.^23^ In addition, plant-based cellulosic fibers contain extractives, such as waxes, fatty alcohols, fatty acids, and various esters, along with other minor constituents like free sugars, starch, pectin, proteins, and mineral salts, which contribute to the surface properties and interactions with other materials.^19,24^ However, it is important to distinguish these minor components from holocellulose, which comprises the combined fraction of cellulose and hemicellulose and typically accounts for 65–70% of a plant's dry weight. Holocellulose is a major structural element of the fiber, not an extractive. It consists of polysaccharides made up of simple sugars—primarily d-glucose, d-mannose, d-galactose, d-xylose, l-arabinose, and d-glucuronic acid—with smaller amounts of l-rhamnose and d-fucose. Due to their abundance of hydroxyl groups, these polymers exhibit strong moisture sorption capacity through hydrogen bonding, playing a critical role in the fiber's hygroscopic behavior.^25^ Cellulose microfibrils are typically 3–5 nm wide when measured individually, but in extracted plant fibers, they often appear as bundles or aggregates ranging from 10–30 nm in width.^26^ These structures, composed of thousands of cellulose molecules, are bound together with lignin and pectin, resulting in a multifiber architecture. The microfibers can be as long as the cell walls, which is many times longer than their width, typically containing 18–24 glucan chains in the cross-section, and are bound together with lignin and pectin.^27^ Hence, the extracted fiber often exhibits a multifiber structure. The width of the fibrils or the diameter of the fibers relies on the amount of matrix, *i.e.*, lignin and pectin, being embedded or degraded after retting or decortication. Higher degradation of lignin and pectin would result in finer fibers or narrower fibers or fibrils. During the matrix (*i.e.*, lignin and pectin) degradation, it may partially break the bond between the fibrils, resulting in a decrease in the fibril width. However, excessive microbial or chemical action can cause degradation of the cellulose fibrils, resulting in a deterioration of the fiber quality.^28,29^

Polysaccharide-based cellulosic fibers are preferred as a sustainable choice over synthetic materials and widely used in composite materials, including hydrogels,^30,31^ fiber-reinforced polymer composites,^32^ human–machine interaction and wearable technologies,^30^ bioelectronics,^33^ and wound-healing applications.^34^ The application of a typical cellulosic fiber (bamboo) in composite materials is illustrated in Fig. 1c, where a cellulosic fiber-reinforced composite hydrogel is developed, inspired by the hierarchical assembly of bamboo.

Cellulose, the dominant structural component, is mainly a semi-crystalline polysaccharide composed of d-glucopyranose units linked by β-(1 → 4)-glucosidic bonds. Its fibrillar arrangement is rich in hydroxyl (–OH) functional groups, initiating hydrogen bonding, which is the main reason for the better strength and inter-fiber interactions.^35^ Hemicellulose, a fully amorphous polysaccharide, is associated with cellulose and comprises low-molecular-weight polysaccharides, including hexoses, pentoses, and uronic acid residues. The presence of hemicellulose in the fiber–matrix influences the moisture absorption, fiber flexibility, and adhesion properties with polymer matrices. Lignin, the third key component, is a highly crosslinked and amorphous aromatic polymer that functions as a natural adhesive within the fiber, and as it is composed of phenylpropanoid units, such as coumaryl alcohol, coniferyl alcohol, and sinapyl alcohol, lignin provides hydrophobicity and resistance to microbial degradation. Due to its structural reinforcement role, it is predominantly concentrated in the middle lamella and secondary cell wall, with nearly 70% of the total lignin content residing in the secondary wall. Pectin, waxes, and water-soluble materials influence the fiber surface characteristics, adhesion potential, and biodegradability.^35^ Pectin is a heterogeneous polysaccharide that provides flexibility and binds microfibrils together. Meanwhile, surface morphology studies reveal that cellulose provides a smooth structural foundation, while hemicellulose and lignin appear as a sticky, amorphous layer that is adhered to cellulose fibrils.^15^

**Fig. 2:**
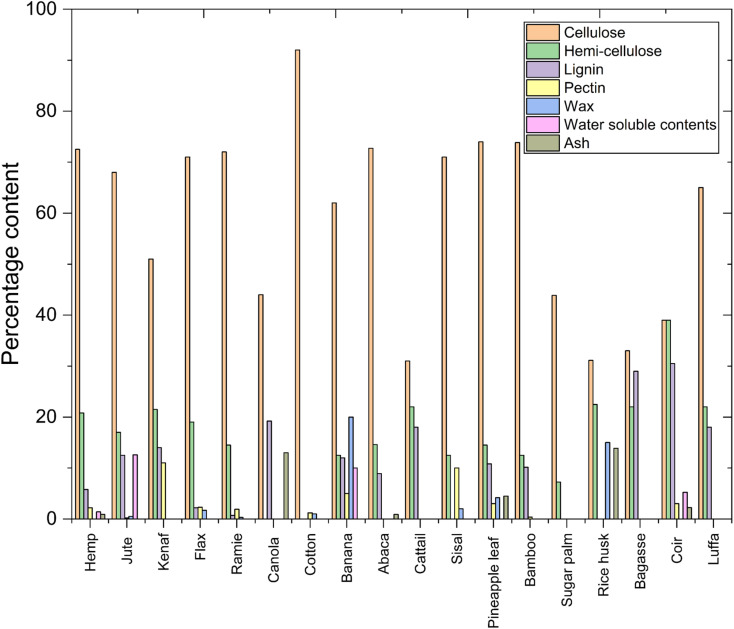
Chemical composition of different cellulosic fibers [data adapted from ref. 36–41 and 44–56].

A comparison of the chemical composition of various natural fibers, indicating their potential for polymer composites, is illustrated in Fig. 2. Cellulose is the primary structural component of plant fibers, and cotton contains the highest proportion—approximately 88–96%—making its fibers highly crystalline and mechanically strong.^36^ Conversely, kenaf (45–57%)^37,38^ and cattail (24–45%)^39,40^ have lower cellulose content and require surface modification to enhance their polymer bonding. Hemicellulose, which influences water absorption and flexibility, is relatively high in coir (32–45%),^41^ possessing a hydrophilic nature. Lignin content, which dictates hydrophobicity and thermal stability, is considerably high in coconut biomass (30.5%).^42^ Thus, it is less prone to water absorption.

Surface content like pectin, wax, water solubility, and ash content are significant in fiber–matrix adhesion. Sisal (10%) possesses high pectin and reduces adhesion efficiency at the expense of enhanced flexibility to hydrophobic resins. Banana (20%)^43^ contains waxes that affect wettability, thus requiring chemical treatment for better integration into polymer matrices. Canola ash content (13%) and rice husk ash content (13.87%)^44^ indicate potential applications for thermal resistance. Surface modification is highlighted above as needed for better fiber adhesion, optimal mechanical properties, and suitable biofibers to develop sustainable composites.

### 2.2 Green chemistry of plant-based cellulosic fibers

**Fig. 3:**
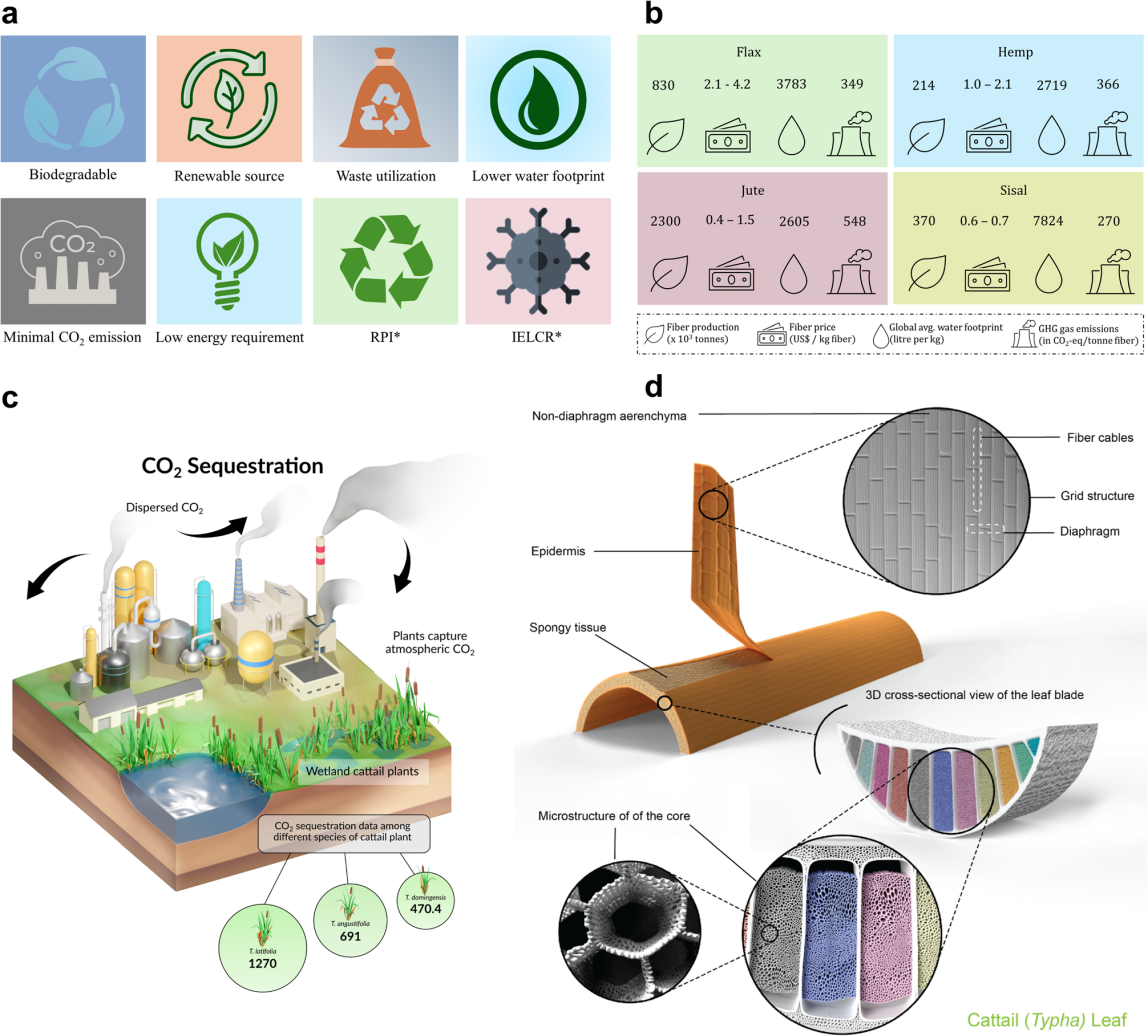
Green chemistry of plant-based cellulosic fibers. (a) Criteria for sustainable fibers; RPI*-recyclability potential index, IELCR*-incremental excess lifetime cancer risk. (b) Overview of the environmental footprints of plant-based cellulosic fibers. (a and b) These figures have been reproduced from ref. 57 with permission from Elsevier B.V., copyright © 2023. (c) Schematic of carbon dioxide sequestration by cattails. The sequestration data is in g CO_2_/*Typha* plant. (d) Macro- and micro-structural models of the lignocellulosic cattail leaf. (c and d) These figures have been reproduced from ref. 58 with unrestricted permission from Wiley-VCH GmbH, licensed under Creative Commons CC BY license, copyright © 2024.

Plant-based cellulosic fibers are often preferred as a greener material choice for manufacturing composites because they are biodegradable, derived from renewable sources, and use discarded materials. The greater recycling potential (RPI) and lesser cancer risk related to carcinogenic chemical exposure contribute to the sustainable attributes of cellulosic fibers (Fig. 3a).^57^ As illustrated in Fig. 3b, certain plant-based cellulosic fibers, such as flax, hemp, jute, and sisal, generally require less water and energy and produce less CO_2_ emissions compared to their synthetic alternatives. However, this is not universally applicable to all natural fibers—for instance, cotton has a notably high water footprint and variable sustainability depending on cultivation practices. An overview of plant-based cellulosic fibers is shown in Fig. 3b, quantifying the fiber production, fiber price, water footprint, and greenhouse gas (GHG) emissions involved during fiber production.

Plant-based cellulosic fibers are typically derived from various plants' stems, leaves, grains, and seed hairs.^32^ Unlike synthetic fibers, these cellulosic fiber crops can potentially facilitate carbon sequestration at different stages of plant growth and reduce carbon emissions, thus preventing climate change and promoting ecosystem resilience. Parvin *et al.*^58^ investigated the carbon dioxide sequestration by cattail plants among their different species (Fig. 3c). Cattail is an emerging lignocellulosic fiber extracted from leaves. Cattails can sequester between ∼470.4 and 1270 g CO_2_ per plant and absorb about ∼1.47 tons of CO_2_ for every 10 tons of dried product to assimilate ∼4 tons of carbon. For a given plant type, the amount of carbon dioxide sequestration depends on the crop or plant growth conditions, growth cycles, number of leaves or stems per plant, water depth, root biomass weight, and dry weight per plant.^58^

Cellulosic fibers are composed of numerous elementary fibrils arranged in complex, irregular morphology. Their surfaces are uneven and textured, contributing to the heterogeneous and porous nature of the overall fiber structure. The cross-sectional structure or the lumen shape varies from circular to polygonal, depending on the type of plant materials used during fiber extraction.^57^ Within a fiber type, cellulosic fibers exhibit variable microstructures due to variations in the cross-sectional shape, internal lumen size and shape, and cell wall thickness. These consequently contribute to the heterogeneity in fiber length, diameter, and strength.^59^ Hence, to date, it is still challenging to quantify the cross-sectional area of plant-based cellulosic fibers and precisely interpret their mechanical properties.

The geometry of the plant's microstructure determines the fiber structure and properties, with variability in the former driving the anisotropic behavior of the latter. Hence, understanding the chemistry and morphology of plant structures alongside fiber structure and properties is essential. The macro- and micro-structural model for the cellulosic cattail leaf, from which the fiber is extracted, is shown schematically in Fig. 3d. This consists of a sandwich structure with an outer bark or epidermis and a spongy core that thickens quadratically toward the center. At the micro-level, they exhibit a hollow and porous structure with diaphragms, partitions, and fiber cables.^58,60,61^

The hollow and porous structure of the plants contributes to the lightweight nature of cellulosic fibers.^57^ The lighter weight of cellulosic fibers makes them ideal for producing lighter composite materials for diversified applications. This is because the specific properties of the composites increase as the fiber density decreases, leading to a decrease in the overall weight of the composite part. Hence, cellulosic fibers with higher specific properties can help to reduce material consumption during manufacturing, compared to materials from fossil-fuel resources, to achieve similar or comparable mechanical performance. This consequently reduces energy consumption and lowers emissions. Secondly, the porous character of cellulosic fibers allows solvents and enzymes to penetrate the fibers, enabling green modification and biodegradation.

## 3. Polymer matrix composites

**Fig. 4:**
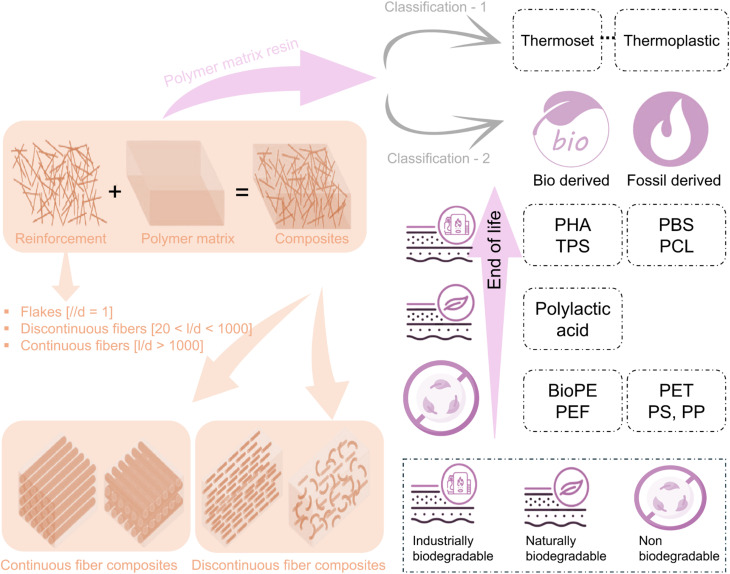
Classification of polymer composites, reinforcing fibers, and polymeric resins. These figures have been reproduced and modified from ref. 63 with unrestricted permission from Wiley-VCH GmbH, licensed under Creative Commons CC BY license, copyright © 2022 and ref. 74 with unrestricted permission from Springer Nature, licensed under Creative Commons CC BY license, copyright © 2023.

**Fig. 5:**
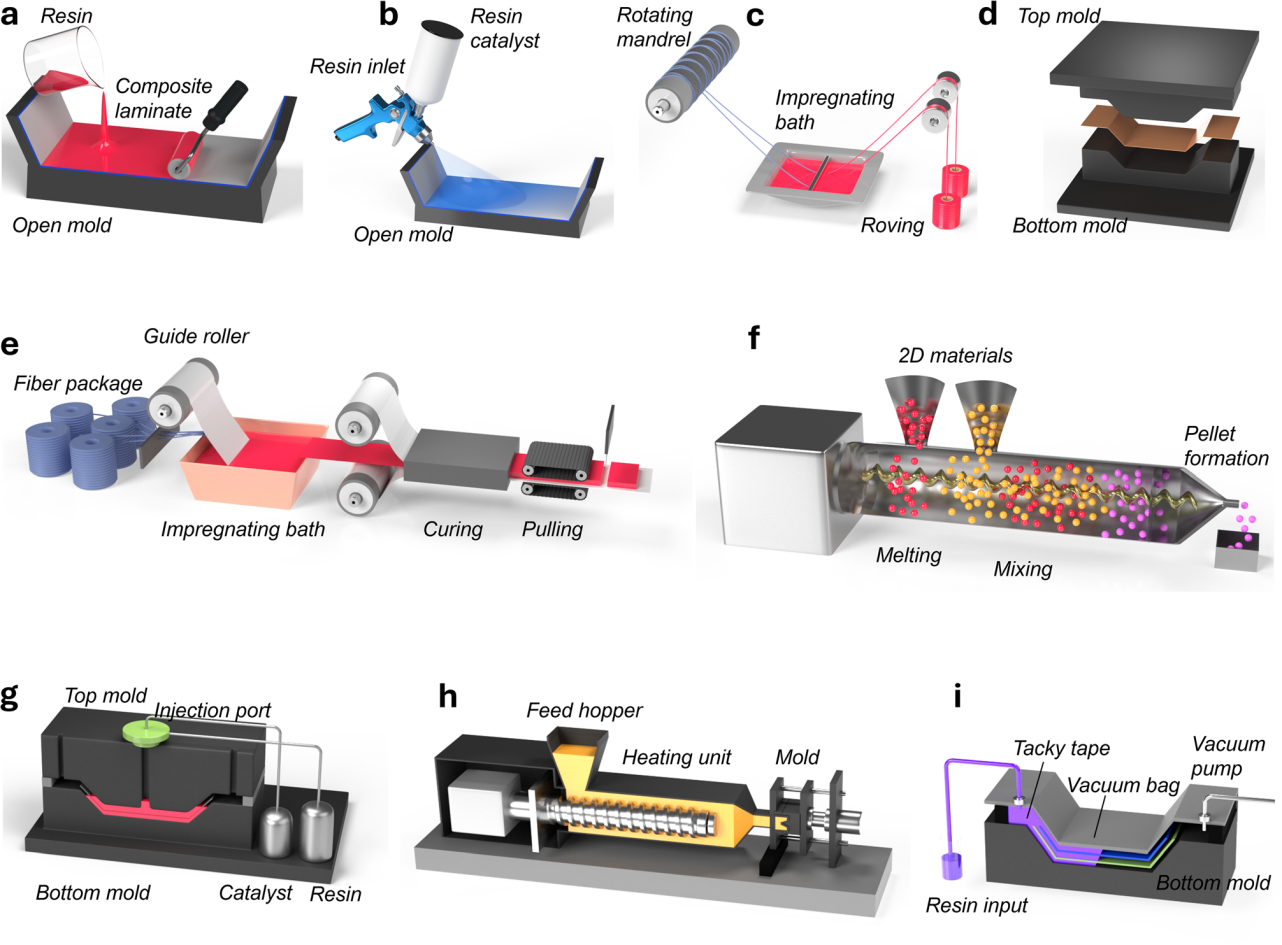
Different manufacturing methods of polymer matrix composites: (a) hand layup, (b) spray layup, (c) filament winding, (d) compression molding, (e) pultrusion, (f) extrusion compound, (g) resin transfer molding (RTM), (h) injection molding, and (i) vacuum infusion.

Composite materials are formed by integrating two or more materials with different properties. The classification of polymer composites and the overview of their parts, such as reinforcing fibers and polymeric resins, are shown in Fig. 4. One of these materials, the matrix or binder, encapsulates and binds together a group of fibers or fragments from a much stronger material, known as the reinforcement.^62^ In biofiber composites, natural fibers like cattail, hemp, flax, and kenaf serve as reinforcement, while polymeric materials (resin) such as epoxy, PLA, and polypropylene act as a binder. The most widely used techniques for manufacturing polymer composites are illustrated in Fig. 5. These include hand layup, spray layup, filament winding, compression molding, pultrusion, extrusion compound, resin transfer molding (RTM), injection molding, and vacuum-assisted resin infusion (VARI).

The hand layup (Fig. 5a) technique is one of the predominant methods for producing open mold polymer composites, entailing the manual placement of reinforcing layers, followed by the application of liquid resin. Hand layup has a low start-up cost and enables diverse product designs; however, this method is limited due to its lower production rate, low and non-uniform reinforcement properties, and dependence on manual precision, making it unsuitable for large-scale and high-performance composite manufacturing.^63–65^

Spray layup (Fig. 5b) and filament winding (Fig. 5c) are also considered as open molding techniques for manufacturing fiber-reinforced composites. While the latter is automated, the former depends on an operator to regulate the preform and composite properties. Filament winding is used for manufacturing axisymmetric components, including pipes, tubes, tanks, vessels, driveshafts, missiles, and pressure vessels. During filament winding, the resin-impregnated continuous fiber is wound onto a rotating mandrel at a predetermined angle, following curing for solidification before the mandrel is removed. It is possible to achieve higher fiber volume fractions (*V*_f_: 60–80%) and greater specific strengths (*E*/*σ*) in filament winding.^66–68^

Compression molding (Fig. 5d), extrusion compounding (Fig. 5f), and injection molding (Fig. 5h) are some of the most widely used closed mold composite manufacturing processes. During compression molding, prepregs are placed into an open heated mold cavity when sandwiched between platens in a hydraulic press. At the same time, in extrusion compounding, fibers and resins are fed into the extruder through a hopper, conveyed forward by a feeding screw (single or twin), and shaped through a 2D die. Similarly, in injection molding, the measured material is injected into a mold through a nozzle following cooling and solidification. Unlike compression molding and extrusion compounding, injection molding can produce more complex 2D composite parts in a wide range of sizes and forms with higher precision and lower processing time.^58,63,66,69,70^

Vacuum infusion (Fig. 5i) or vacuum-assisted resin transfer molding (VARTM) is an improved version of the RTM process (Fig. 5g). In VARTM, the resin is injected into the mold under vacuum pressure (−ve 15 psi), where the dry preforms (non-woven or woven) are manually laid on the gel-coated or non-stick release-coated tool surface. Cores, if used, are placed at the required locations and secured to the preform with tackifiers. A flow (*i.e.*, distribution) medium that helps in the flow of resin may be used, which is typically stacked onto the preform or sandwiched between two layers of preform.^71–73^

The above fabrication techniques are often used to manufacture cellulosic fiber-reinforced polymer composites. Palm leaf,^75^ jute,^76^ flax,^70^ and rice husk^77^ are mixed with polypropylene, epoxy, soy protein, and PLA to create environmentally friendly composite materials. The production routes, including injection molding, compression molding, resin transfer molding, hand lay-up, melt blending, and resin infusion, determine these composites' final mechanical performance and interfacial adhesion. Compression molding, used in jute/epoxy^76^ and bamboo/epoxy^78^ composites, allows for good matrix–fiber bonding, whereas hand lay-up, used in rice husk/epoxy^77^ and coir fiber/epoxy,^79^ offers low-cost manufacturing with average mechanical properties.

These findings reflect the influence of the fiber orientation and fabrication processes on the composite performance. Resin infusion in flax fiber/epoxy^80^ enhances fiber wetting to improve interfacial bonding. Melt mixing in kenaf fiber/PLA^81^ enables uniform dispersion of the fibers for maximum composite properties. By comparing these processes, researchers can rationally optimize the manufacturing processes to increase the adhesion, toughness, and mechanical properties to create innovative biofiber composites suitable for sustainable industrial applications.

### 3.1 Polymeric matrix/resin

The polymeric matrix typically associates stress transfer, fiber bonding, and protection against mechanical and environmental degradation. For reinforcing fiber contents to bear load effectively, the matrix possesses a lower modulus and higher elongation than the fibers. This equilibrium ensures that stress has been efficiently transferred from the matrix to the fibers; thus, the composite maintains its structural integrity under mechanical loads. Additionally, the matrix can influence the service temperature and processing parameters for composite manufacturing, influencing the performance and applicability of the composite materials.^82^ Resins used in composites are broadly classified into thermoset and thermoplastic polymers. Thermoset resins start as liquids or low-melting solids and undergo curing *via* a catalyst, heat, or both, forming a rigid, cross-linked structure. Once they are cured, they become insoluble and infusible, and then they cannot undergo any further reshaping or remelting. Although thermosets exhibit excellent thermal stability, chemical resistance, and structural rigidity, they can never be recycled due to the irreversible curing process.^82^

In contrast, thermoplastic resins typically soften upon heating, enabling the process for molding and reshaping, and regain rigidity upon cooling. As they do not have any permanent chemical cross-linking, thermoplastics are often considered less suitable for high-performance structural applications, but remain advantageous in recyclability and processability.^83^ Most resins used in composite manufacturing include unsaturated polyesters, epoxies, and vinyl esters, which provide a balance between mechanical strength, adhesion, and durability. Other resin types, such as polyurethanes and phenolics, are used in specialized applications, but are less prevalent due to processing limitations.^83^Fig. 4 illustrates the classification of resins, highlighting their common examples and applications. The selection of resin is critical in the process as it determines the composite processing methods, service life, performance, and environmental resistance. While the reinforcing fibers primarily govern the stiffness and mechanical strength, the matrix dictates the maximum service temperature, manufacturing feasibility, and long-term durability.^84^Table 1 presents the properties of various thermoset resins, emphasizing their role in enhancing biofiber-based composite performance. The optimal resin selection is crucial in biofiber-reinforced composites, ensuring compatibility with natural fibers, while maintaining mechanical integrity, environmental resistance, and manufacturability in diverse engineering applications.^84^

**Table 1:** Characteristics and applications of various thermoset resins for biofibers^a^

Resin	Description	Properties	Applications	References
EP	Synthesized from the reaction of a dihydric phenol, like bisphenol-A, with excess epichlorohydrin in an alkaline medium	Low cure shrinkage, better dimensional stability, adhesion, resistance to corrosive liquids and environments, not UV resistant	High-performance composites, filament-wound composites, circuit board encapsulants	85 and 86
DAP	Improved version of polyester resin using diallyl phthalate (DAP) monomer	Low vapor pressure, less environmental pollution, lowest post-cured shrinkage, resistance to moisture	Electrical and electronic applications	87
VE	Produced from the condensation reaction between phenol(s) and formaldehyde	Excellent toughness and chemical resistance	Sheet and bulk molding compounds are used in satellite plants, bulk molding compounds for electrical parts, and housings	88 and 98
PH	Produced from the condensation reaction between phenol(s) and formaldehyde	High thermal and chemical stability	Carbon–carbon composite materials	89 and 99
CE	Produced by reacting halogenated cyanide with phenolic compounds under alkaline conditions	High-temperature stability, low dielectric constant, deficient moisture uptake, and micro-crack resistance	Microelectronics, aerospace composites	90
p	High-temperature PMR (polymerization of monomer reactants) resins are preferred over epoxy for composites	Thermo-oxidative stability, high processability	Used in high-temperature polymer matrix composites	91 and 92
BMI	Produced *via* vinyl-type polymerization of the pre-polymer with two maleimide groups	High-strength, high-temperature performance, good retention of mechanical properties, fire, and chemical resistance, high thermal and high oxidative stability	Aerospace structures, printed circuit boards, structural laminates	82, 92, 93 and 100

a EP = Epoxy, DAP = Diallyl phthalate-based resin, VE = Vinyl ester, PH = Phenolic, CE = Cyanate ester, P = Polyimide, BMI = Poly(bismaleimide).

Resins have played a vital role in enhancing mechanical strength, thermal stability, and adhesion, thereby improving the composite materials' overall performance and durability. Epoxy (EP) resins, manufactured from dihydric phenols like bisphenol-A and epichlorohydrin,^85,86^ offer low cure shrinkage, good adhesion, and corrosion resistance, and are ideal for high-performance composites and encapsulants of circuit boards. Similar diallyl phthalate-based (DAP) resins, as an improved version of polyester,^87^ offer low vapor pressure and zero post-cure shrinkage, and hence prove effective in electrical use. Among phenolic-based resins, vinyl ester (VE)^88^ resins show high chemical and toughness resistance, while phenolic (PH) resins^89^ display high thermal and chemical stability, suitable for carbon composites. Cyanate ester (CE) resins^90^ show micro-crack resistance and thermal stability at high temperatures, making them ideal for aerospace and electronics. Polyimide (P) resins^91,92^ have uses in high-temperature polymer matrix composites, whereas bismaleimide (BMI) resins^92,93^ have high strength and thermal resistance, and are employed in structural aerospace materials.

The sustainability of plant-based cellulosic fiber composites is limited due to the widespread utilization of synthetic polymeric resins. Hence, the vegetable oil-based bio-epoxy resins have recently gained attention as alternatives to synthetic petroleum-based resins. Vegetable oils, such as mustard oil, soybean oil, castor oil, and canola oil, contain unsaturated fatty acids. These oils undergo epoxidation *via* the oxidation reaction of hydrogen peroxide and peracids to produce epoxy-functionalized oil-based resin.^94^ Furthermore, the plant-fiber component itself, particularly lignin, has been increasingly studied to assess its feasibility as a bio-based polymeric resin for films and composites.^95^ Lignin can be transformed into phenolic, as well as epoxy-based resins.

While the thermoplastic composites can be recycled, composites manufactured using thermoset resin are typically difficult to recycle and reuse because of their infusible and insoluble covalently cross-linked three-dimensional networks.^96^ However, with growing environmental concerns, more research is now emphasized on studying the closed-loop recycling mechanisms of thermosets. The two primary techniques for the closed-loop recycling of thermosets are hydrolysis and de-cross-linking of the thermoset composites. The hydrolysis mechanism is typically conducted in an acidic, alkaline or catalyst solution and the de-cross-linking occurs *via* the exchange reaction of dynamic bonds. The rate of degradation and recovery during the hydrolysis and de-cross-linking process strongly relies on the structure and wettability of the thermoset resin, as well as the wettability of the depolymerization solution.^96,97^

### 3.2 Adhesion theories: fiber–resin interaction in composite materials

The strength of composite materials is critically dependent on fiber–matrix adhesion, which determines the efficiency of stress transfer between the matrix and the reinforcing fiber. This adhesion is influenced by mechanisms such as adsorption and wetting, electrostatic attraction, chemical bonding, and mechanical interlocking.^1,101–103^ Adhesion theories are complex, making the attribution of bonding to a single mechanism hard. Usually, multiple mechanisms are involved, and their importance varies between adhesive systems. Mechanical interlocking, an early and straightforward explanation for adhesive bonding, suggests that adhesives bond by penetrating the irregularities on the adherend surface. This understanding led to the practice of roughening surfaces to enhance adhesion.

**Fig. 6:**
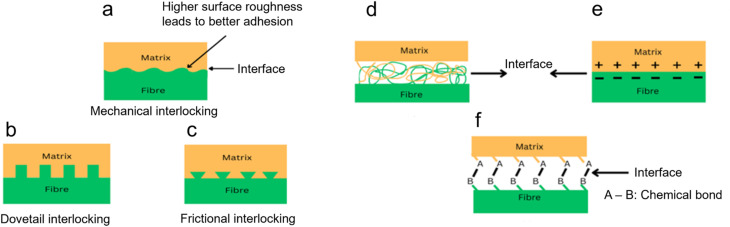
(a–c) Schematic of the mechanism of mechanical interlocking (a) and two different types of mechanical interlocking: (b) dovetail interlocking and (c) frictional interlocking. (d–f) Schematic of traditional fiber–matrix interfacial bonding mechanisms: (d) interdiffusion, (e) electrostatic adhesion, and (f) chemical bonding. This figure has been reproduced and modified from ref. 110 with permission from Elsevier, copyright © 2016.

The mechanism of mechanical interlocking is shown in Fig. 6a–c, and the mechanism of other adhesion theories is shown in Fig. 6d–f. Interlocking mechanisms in composite materials enhance adhesion between the matrix and fiber through structural modifications that prevent separation under stress. As Kalu *et al.*^104^ described, mechanical interlocking relies on nanoscale sculpturing to create micro-mechanical interlocking sites, improving adhesion in hybrid materials. This technique is particularly beneficial in polymer and ceramic composites, where surface roughness increases contact and prevents fiber slippage. Dovetail interlocking involves matrix protrusions shaped like dovetails that fit into fiber indentations, enhancing shear resistance and mechanical stability.^105^ This approach is widely used in biomedical and aerospace applications to improve interfacial bonding. Frictional interlocking utilizes triangular matrix protrusions that engage fiber indentations and relies on frictional forces to maintain adhesion.^106^ The three traditional fiber–matrix interfacial bonding mechanisms, interdiffusion, electrostatic adhesion, and chemical bonding, are important mechanisms for enhancing the composite material performance. Interdiffusion occurs when the matrix and fiber polymer chains diffuse into each other, forming a physically entangled interface to strengthen the adhesion and mechanical stability. This is a prevalent mechanism in natural fiber composites, with specific emphasis on its role in maximizing fiber–matrix interaction.^107^ Electrostatic adhesion relies on the interplay between oppositely charged matrix and fiber surfaces, leading to a strong bonding force within the interfacial area. Lee *et al.*^15^ studied this mechanism in plant-fiber-reinforced polymers and demonstrated its significant role in stress transfer enhancement. Chemical bonding appreciates hydrogen or covalent bonds formed between functional groups in the fiber and matrix, resulting in a stable and long-term interface. Amiandamhen *et al.*^108^ provided an overview of the impact of chemical treatment on the fibers' composition and showed how treated surfaces enhance adhesion between the matrix and fiber. All these bonding processes play a role in the composite materials' mechanical properties, durability, and environmental resistance, and make them pivotal in aerospace, automobile, and biomedicine engineering applications.

Mechanical interlocking has been particularly effective for fibrous substrates like papyrus, paper, leather, and wood, where the adhesives can penetrate the fibers and solidify. However, this principle is not universally applicable. For instance, increasing surface roughness in wood specimens can decrease the bond strength.^109^

Cellulosic fiber and polymeric matrix adhesion chemistry in composites is governed by several theories of adhesion, summarized in Table 2, each of which contributes uniquely to the structural integrity and performance of the composite. Electrostatic adhesion plays a role in those composites in which charge interaction enhances the fiber–matrix adhesion, for example, in electrostatic discharge or electrospinning treatments. Chemical adhesion, *via* covalent bonding, is critical for the strong interfaces between cellulosic fibers and polymers, and is usually complemented by chemical modifications, which introduce reactive functional groups. Physical or interdiffusion adhesion relies on surface energy and wettability, wherein treatments like alkaline modification regulate the fiber polarity for improved bonding. Mechanical interlocking, *via* surface roughness and fiber porosity, generates strong adhesion by physical entrapment of polymer matrices. Weak boundary layers (WBL) due to impurities or surface degradation can be reduced by employing suitable surface preparation methods, thereby ensuring composite integrity. Acid–based interactions, led by surface energy models, continue to sophisticate adhesion chemistry by optimizing fiber–matrix interactions through chemical treatments.

**Table 2:** Comprehensive analysis of different adhesion theories

Theory of adhesion	Mechanism	Factors for good adhesion	Enhancement methods	Examples/evidences	References
Electrostatic adhesion	Adhesion due to electrostatic forces between materials, acting like plates of a condenser	Presence of opposite charges, interatomic and intermolecular forces, and ionic state of plant fibers	Electrostatic discharge treatment to induce charges, electrospinning process to create electrostatic fibers	Integration of electrostatic fibers like carbon/glass for stronger bonding H^+^, O^2−^	66
Chemical adhesion	Adhesion through covalent bonds, where two atoms share an electron pair	Chemical bonding sites, hydrophobicity of fibers, and intensity of chemical bonding	Chemical modifications to increase bonding sites, use of hydrophobic chemical treatments, and increased chemical bonding sites through modifications	Removal of OH^−^ and substitution with hydrophobic groups, such as acetylation, to improve bonding with hydrophobic resin	114–116
Physical or interdiffusion adhesion	Adhesion through atomic and molecular interactions is influenced by surface tension and free energy	Surface energies, similar surface polarities with the matrix, good wettability	Alkaline treatment to modify surface energy, use of maleated coupling agents, and regulate surface energies and polarities through surface treatments	Alkaline-treated coir fibers with better wettability and higher work of adhesion	117
Mechanical interlocking	Adhesion through physical interlocking *via* pores, gaps, or irregularities	Surface roughness, penetration of polymer into fiber structures, and wettability	Alkaline treatment to create a rough surface, removal of non-cellulosic components to increase penetration, ensuring clean and rough fiber topography for better resin flow	A rough fiber surface provides more anchor points for mechanical interlocking, polymer resin flows into lumens and pores for better mechanical interlocking	118–121
Weak boundary layers (WBL)	Adhesion issues caused by weak boundary layers, like impurities, air bubbles, or surface damage	Weak boundary layers result in lower mechanical strength and potential adhesion failure at the interface	Proper surface preparation techniques, use of adhesives that can tolerate weak boundary layers	Moisture-tolerant adhesives with isocyanate functionality that react with water to form urea linkages	122
Acid–base theory	Adhesion through acid–base interactions, where an acid bonds to a base by sharing an electron pair	Acid–base interactions, surface free energy models, polar *vs.* non-polar substrates	Adjustment of surface energy, improving acid–base interactions through chemical treatments	Zein treatment has been shown to enhance the basic character of kenaf, agave, and hemp fibers, while diminishing it in agave hybrid, flax, pineapple, and sisal fibers, and alkaline treatment decreases the basic character for all fibers except kenaf	123 and 124

The mechanisms described by these adhesion theories can be used to understand the relationship between adhesion chemistry and mechanical properties, such as the mechanical strength, durability, and environmental resistance of polymer composites. For instance, acid–base interactions have been shown to enhance interfacial shear stress and thus practical adhesion.^111^ Chemical treatments, such as silane or acetylation, that modify fiber surfaces to increase compatibility with polymer matrices, lead to composites with better mechanical properties.^112^ The interplay between the mechanisms of adhesion and fiber–matrix chemistry controls the composite's resistance to imposed stresses, water absorption, and temperature changes.^113^

### 3.3 Weak boundary layers

**Fig. 7:**
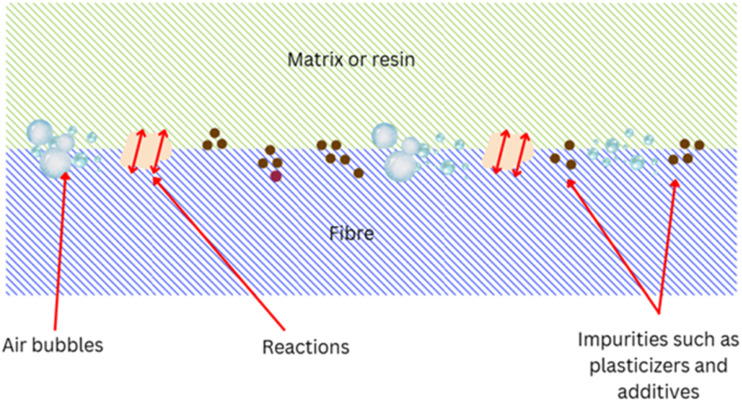
Different types of weak boundary layers in composites.

Fig. 7 demonstrates the different types of weak boundary layers in polymer composites. The composite material weak boundary layers significantly impact the adhesion and mechanical properties, and come from both mechanical and chemical sources. Mechanical weak boundary layers include air bubbles introduced during processing, machining-induced surface damage, and dust or debris contamination, which all reduce adequate bonding. Chemical weak boundary layers result from lubricant contamination, plasticizers, additives, old surfaces or inactive surfaces, and environmental weathering, producing weakened interfacial strength.^123^ Bikerman^125^ classified weak boundary layers according to their phase origins into air, adhesive, adherend, and mixtures thereof, including air-adhesive, air-adherend, adhesive-adherend, and all three together. Recent studies have addressed the detection and prevention of weak boundary layers in composite structures. Bu *et al.*^126^ employed nonlinear ultrasonic-guided waves to identify localized damage and degradation in bonded composite structures, demonstrating the impact of adhesive layer degradation on mechanical stability. In addition, Yuanwu *et al.*^127^ studied the influence of volume fractions and boundary conditions on the computed effective material properties of Al/Ni composites, emphasizing the bonding strength improvement caused by modified microstructures. These low-strength boundary layers must be well understood to achieve the maximum composite longevity and prevent premature failure in aerospace, automotive, and structural applications. Surface alteration techniques like plasma treatments and nano-coatings are being explored to enhance fiber–matrix bonding and mitigate the effects of low-strength boundary layers.

## 4. Effect of fiber structure, architecture, and morphology on adhesion

Table 3 depicts the crucial biofiber characteristics that directly influence adhesion chemistry in polymer composites, including beneficial and problematic properties. These properties include physical characteristics, such as fiber length, diameter, surface roughness, and chemical composition. Surface roughness and fibrillation are essential for mechanical interlocking and improving the fiber–matrix interaction.^128–130^ Fibrillated fibers, through a larger contact area, enable improved adhesion, increasing the overall composite durability. Micropores serve positively by optimizing the contact surface area and enhancing mechanical bonding.^131^ Moderate lignin content, though complex in adhesion behavior, has also positively impacted cotton/PLA composites by stabilizing the matrix and improving compatibility.^132,133^ On the other hand, hydrophilicity and water uptake tend to cause fiber swelling, disrupting adhesion and mechanical property degradation.^134,135^ In the same way, waxes, oils, and pectin hinder fiber interlocking as they coat reactive functional groups, lowering the bonding efficiency.^15,132,136^ A reduction in the fiber diameter facilitates stress transmission but increases moisture absorption, which may compromise adhesion.^15,137^ Biofiber composites can be made more effective and durable for structural applications by optimizing the fiber's roughness, micropores, and fibrillation through surface treatments, regardless of limitations.

**Table 3:** Impact of cellulosic fiber properties on adhesion

Property	Impact on adhesion	References
Hydrophilicity and water absorption	Causes swelling, compromising adhesion interface and reducing mechanical properties	134 and 135
Amorphous regions	High affinity to moisture reduces compatibility with the hydrophobic matrix	135
Waxes and pectin	Obstructs interlocking by covering reactive functional groups	132 and 136
Lignin	Relationship with adhesion is complicated. It obstructs interlocking by covering reactive functional groups. Moderate lignin content was found to have a positive impact when added to cotton/PLA composites	132, 133 and 136
Surface roughness	Promotes stronger mechanical interlocking and increases the interaction between fiber and polymer matrix	128 and 129
Fibrillation	Increases contact area with matrix, promoting adhesion	130 and 138
Wax and oils	The presence of these compounds obstructs interlocking by covering reactive functional groups, reducing adhesion	1, 139 and 140
Fiber porosity (lumen)	Might not have a detrimental impact on the adhesion since it does not affect stress concentrations or fiber/matrix debonding but may absorb moisture	141
Diameter	Smaller diameter fibers improve stress transmission and load distribution, but have higher moisture absorption and swelling tendency, which can damage interfacial bonds	15, 137 and 142
Micropores	Enhances the contact area and mechanical interlocking	131
Length	Fibers shorter than the critical length: higher interfacial adhesion, but reduced mechanical performance	143 and 144

### 4.1 Moisture absorption, diffusion, and adhesion behavior of composites

Natural polymers such as cellulose, hemicellulose, and lignin contain numerous hydroxyl (–OH) groups. The hydroxyl groups are polar in nature and readily form H-bonding with water molecules, leading to water absorption and increased polarity of the fiber surface. This affinity renders the fibers hydrophilic (*i.e.*, to attract and retain water) and polar, which affects their chemical compatibility. When mixed into hydrophobic polymer matrices, this polarity mismatch results in poor interfacial adhesion, weakening the mechanical integrity of the composite material. The hydrophilicity of such fibers is also responsible for dimensional instability and humid-weather degradation, both of which make it less convenient for use in polymer composites. Research has determined that chemical treatment or the use of a compatibilizer is usually needed to improve the fiber-to-matrix bond and enhance the composite performance.^145–147^

**Fig. 8:**
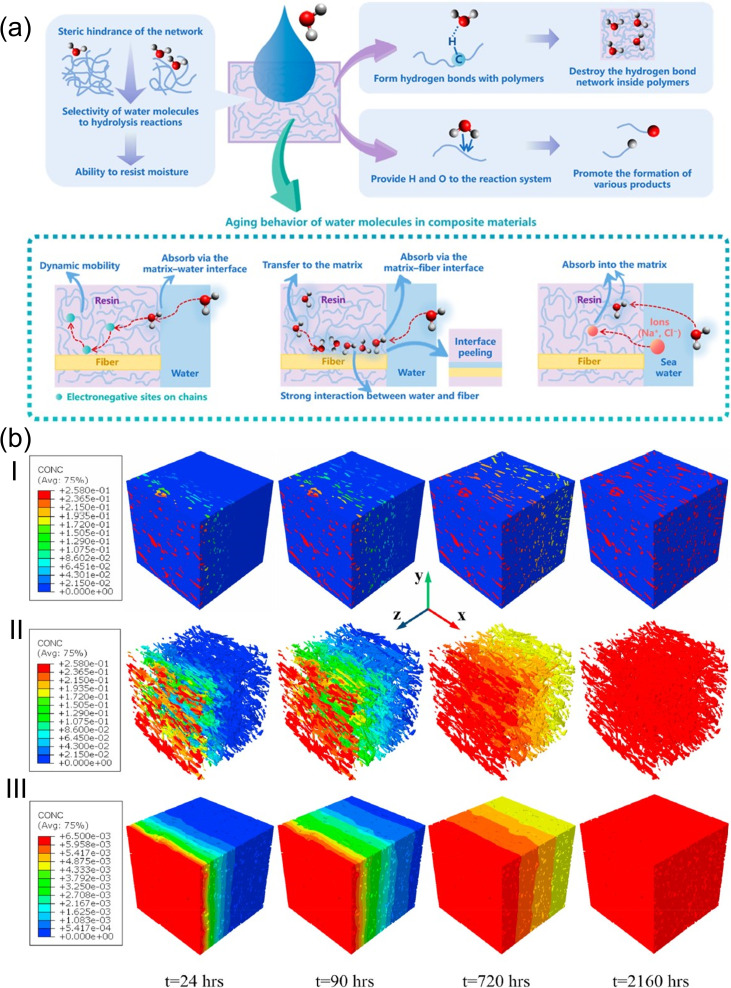
(a) Aging mechanisms of polymer composites under the influence of moisture. This figure has been adapted from ref. 148 with unrestricted permission from MDPI, licensed under Creative Commons CC BY license, copyright © 2023. (b) Transient moisture distribution within the (I) jute/PLA composite, (II) jute fiber, and (III) PLA matrix at various aging times. This figure has been adapted from ref. 154 with permission from Elsevier Ltd, copyright © 2020.

**Fig. 9:**
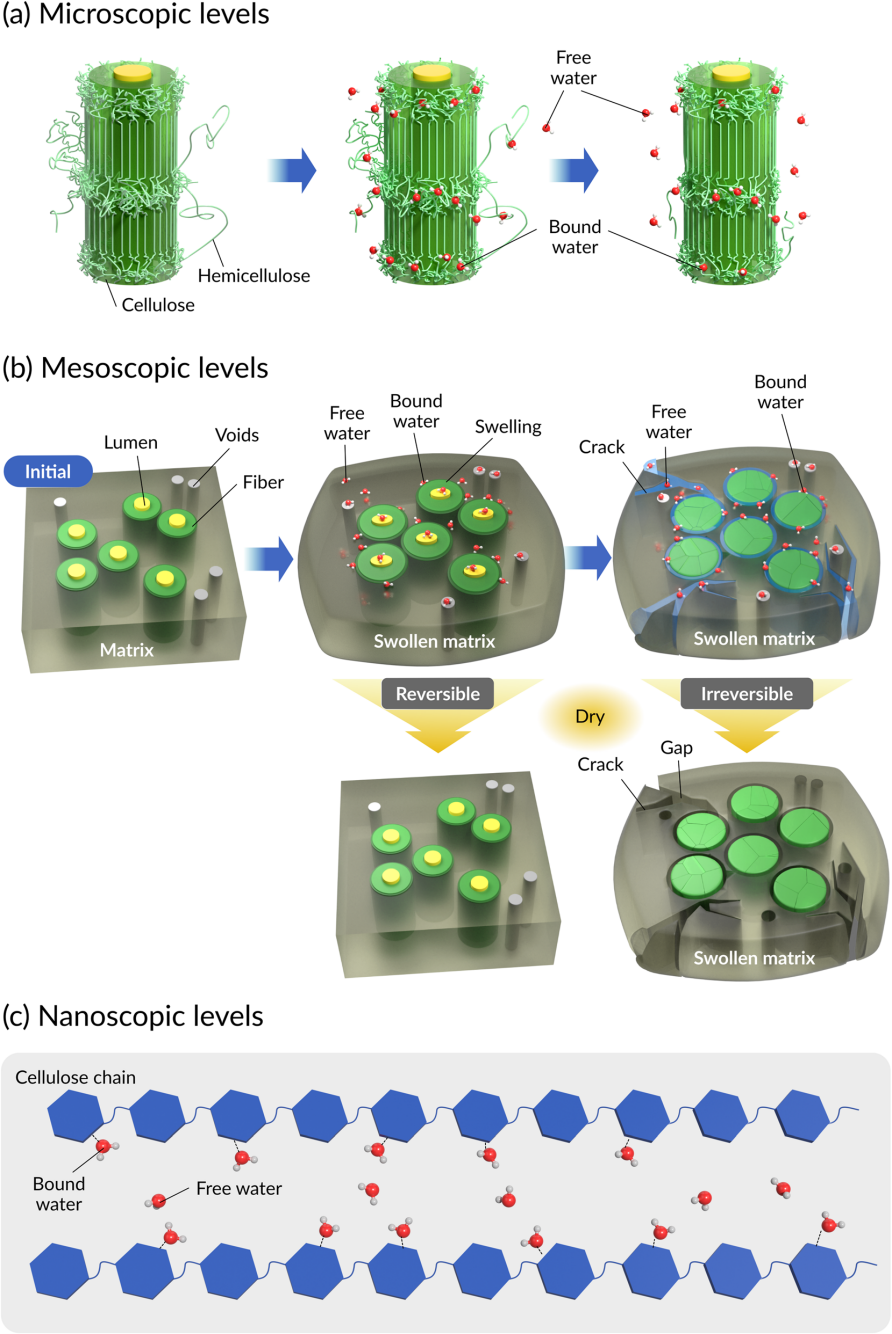
Water absorption and degradation mechanisms of cellulosic fiber-reinforced polymer composites. (a) Microscopic levels, (b) mesoscopic levels, and (c) nanoscopic levels.

The generic aging mechanisms of polymer composites under the influence of moisture are illustrated in Fig. 8a.^148^ Moisture affects the structure, properties, and aging behavior of composite materials. The activation energy of thermal degradation decreases with the increase in the water content, while the pre-exponential factor increases.^148,149^ Also, the water absorption and degradation mechanisms of cellulosic fiber-reinforced polymer composites are illustrated in Fig. 9a–c. The moisture absorption kinetics in fiber-reinforced polymer composites during degradation can be modeled using eqn (1), where *M*_m_ and *M*_*t*_ are the % moisture regained at equilibrium and at a given time *t*, respectively. *k* and *n* are constants related to the mechanism of moisture diffusion. *k* points to the interaction intensity between the polymer and water, and *n* reflects the mode of diffusion.^71,150^1
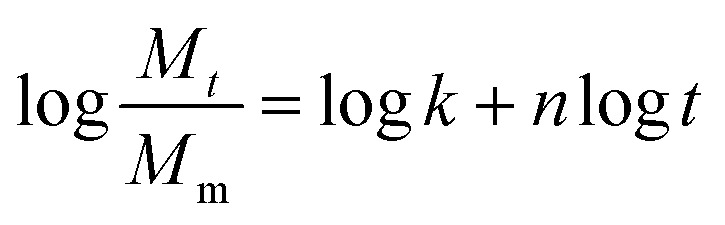


The diffusion coefficient (*D*) can be determined using Fick's law,^71,150^ as per eqn (2), where *h* corresponds to the thickness of the composite samples and *θ* corresponds to the slope of the initial linear portion of the moisture absorption curves.2
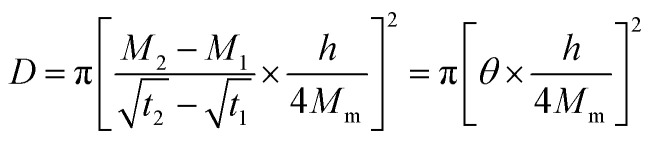


Moisture and temperature are often considered together with moisture absorption during the degradation process, as materials typically undergo hygrothermal aging during usage and storage.^148^ During the hygrothermal aging process of polymers, water molecules affect the stability of polymers by forming hydrogen bonds with the polymers or participating in reactions. The mobility of the molecular chains increases with the increase in moisture content due to the cleavage in the hydrogen bonding between water molecules and polymer chains.^151^ Also, during the aging or degradation process, the crosslinked thermoset polymer undergoes protonation of the water molecule and nucleophilic attack on the C–O bond of the ether linkages.^152^

Cellulosic fiber polymer composites are assumed to be 100% dense composites after manufacturing. However, these composites can absorb water at the micro- and nano-scale after long-term aging due to micro- and nano-scale pores and hydrophilicity of the reinforcing materials. The water types adsorbed by cellulosic plant fibers are bound water and free water, where the bound water is strongly immobilized by adsorption, while the free water is physically entrapped.^153^ Water molecules frequently form H-bonds with cellulose structures, disrupting the H-bond networks and causing crack formation, leading to degradation. These consequently reduce the mechanical strength and aging resistance of cellulose. In summary, the micro- and nano-level hierarchical structures and properties of the reinforcing fibers accelerate the water-based degradation of composites. Polymer matrices are hydrophobic. Hence, they do not contribute to water absorption. An increase in the polymer matrix content or a decrease in the fiber content will reduce the overall water absorption and prevent degradation. However, a decrease in the fiber fraction will result in a reduction of the overall mechanical performance of the composites. For a given fiber fraction, an increase in the percentage of cellulose in the fiber will cause a reduction in water absorption, given that only the percentage of non-cellulosic component present in the fiber accounts for water absorption. Hence, surface engineering is the key to regulating water adsorption, while keeping the optimal fiber fraction for better composite properties (mechanical).

Jiang *et al.*^154^ investigated the water diffusion behavior of short jute fiber-reinforced composites using a 3D finite element modeling approach (Fig. 8b), where the volume fraction, dimension, and orientation distribution of fibers and composites were acquired by segmentation of X-ray computed tomographs. Water initially saturates the surface regions of the PLA matrix and jute fibers upon contact, subsequently diffusing toward the symmetric center, with early-stage non-uniformity caused by the clustering of jute fibers and anisotropic diffusion. Typically, the rate of diffusion of water molecules is higher in fibers than in neat polymer resins, while the diffusion in composites lies in between. For a given fiber orientation, diffusion in a polymer composite increases with the increase in fiber volume fraction. Hence, surface treatment can be employed to reinforce fibers to limit the diffusion of water molecules and obtain optimal interfacial bonding in composites.

### 4.2 Effect of chemical composition on the adhesion behavior

**Fig. 10:**
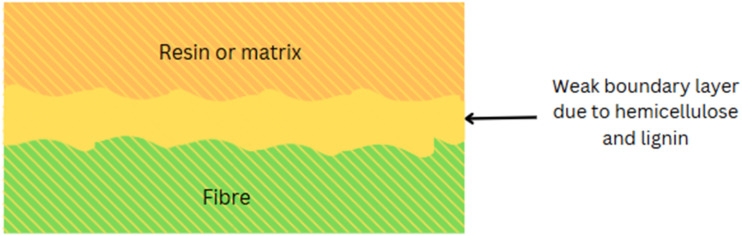
Formation of the weak boundary layer by hemicellulose and lignin.

The hemicellulose and lignin form a weak boundary layer between the cellulose and the matrix (Fig. 10), which is not conducive to adhesion.^108^ Formation of weak boundary layers in composite materials is dominated by hemicellulose and lignin, affecting fiber–matrix adhesion and mechanical behavior. Hemicellulose is a water-soluble polysaccharide that swells when it absorbs water, degrading the fiber–matrix interface. Lignin is a three-dimensional aromatic polymer that forms a hardened, brittle interface that limits stress transfer from fibers to the matrix. These factors augment poor adhesion and mechanical instability. The thin boundary layer results in low load-carrying capacity, high delamination, and reduced durability of the composite materials, as indicated by the photograph, where the matrix and fiber show poor bonding. These effects reduce mechanical performance, durability, and load transfer. Current studies^155–157^ demonstrate how excessive lignin prevents successful fibrillation and hemicellulose increases hygroscopicity, both of which reduce adhesions.

Huber and Müssig^158^ investigated the adhesion and interfacial shear strength (IFFS) between natural fibers—flax, hemp, and cotton—and polymer matrices, specifically polypropylene with a coupling agent (maleic anhydride grafted polypropylene, MAPP) and polylactic acid (PLA), using the single fiber fragmentation test. Their findings revealed that flax had the highest IFFS value of 7.09 N mm^−2^ for fibers with the same diameter, followed by hemp with 6.13 N mm^−2^. Despite having a high cellulose content of 92.7%,^159,160^ cotton exhibited a significantly lower IFFS value of 0.664 N mm^−2^. Flax, with about 62% cellulose content, and hemp, containing approximately 67% cellulose, showed better adhesion with MAPP compared to lignin and pectin-free cotton fibers.^159,160^ This superior adhesion can be attributed to the surface structure and unique chemical composition of cotton fibers, which consist solely of cellulose, hemicellulose, and wax with no lignin and pectin present. In another study, depositing 5–6% bacterial cellulose on sisal and hemp fibers during fermentation significantly improved adhesion with polymer matrices like polylactic acid and cellulose acetate butyrate.^161^ Graupner *et al.*^129^ examined the fiber/matrix adhesion of regenerated cellulosic fibers (lyocell) and bast fiber bundles (flax, kenaf) using PLA, PP, and MAPP resins. They discovered that cellulosic fibers demonstrated higher apparent interfacial shear strength (IFSS) values in PLA and MAPP compared to PP, due to the higher polarity of PLA and MAPP. Bast fibers, containing 2–19% lignin, showed higher IFSS values than lyocell fibers, which lack lignin.^129^ Furthermore, incorporating lignin into cotton/PLA composites has been shown to significantly improve the connection between fibers and the matrix and between individual fiber layers in a multilayer web, thereby reducing delamination and enhancing overall adhesion.^133^ Jute strands containing 4% lignin were identified as the most suitable reinforcement for PLA. A lignin content of lower or higher than 4% resulted in a reduced IFFS compared to the 4% lignin content.^162^ Nevertheless, further research is needed to determine the optimal lignin content or combination to enhance bonding in composite materials across all biofibers. To evaluate the effectiveness of the waxy layer on interfacial bonding, coconut fibers with and without the waxy layer, and those modified with an isocyanate derivative of cardanol (CTDIC), were tested. Wax-free fibers were produced by soaking them in a 5% sodium hydroxide solution for 84 hours at room temperature without degrading the tensile properties. The natural waxy layer provided superior bonding compared to the grafted C15 alkyl chain, as its removal doubled the critical fiber length and reduced the tensile strength by 40% and modulus by 60% (Brahmakumar *et al.*, 2005).^163^

### 4.3 Effect of fiber length on the adhesion and composite properties

**Fig. 11:**
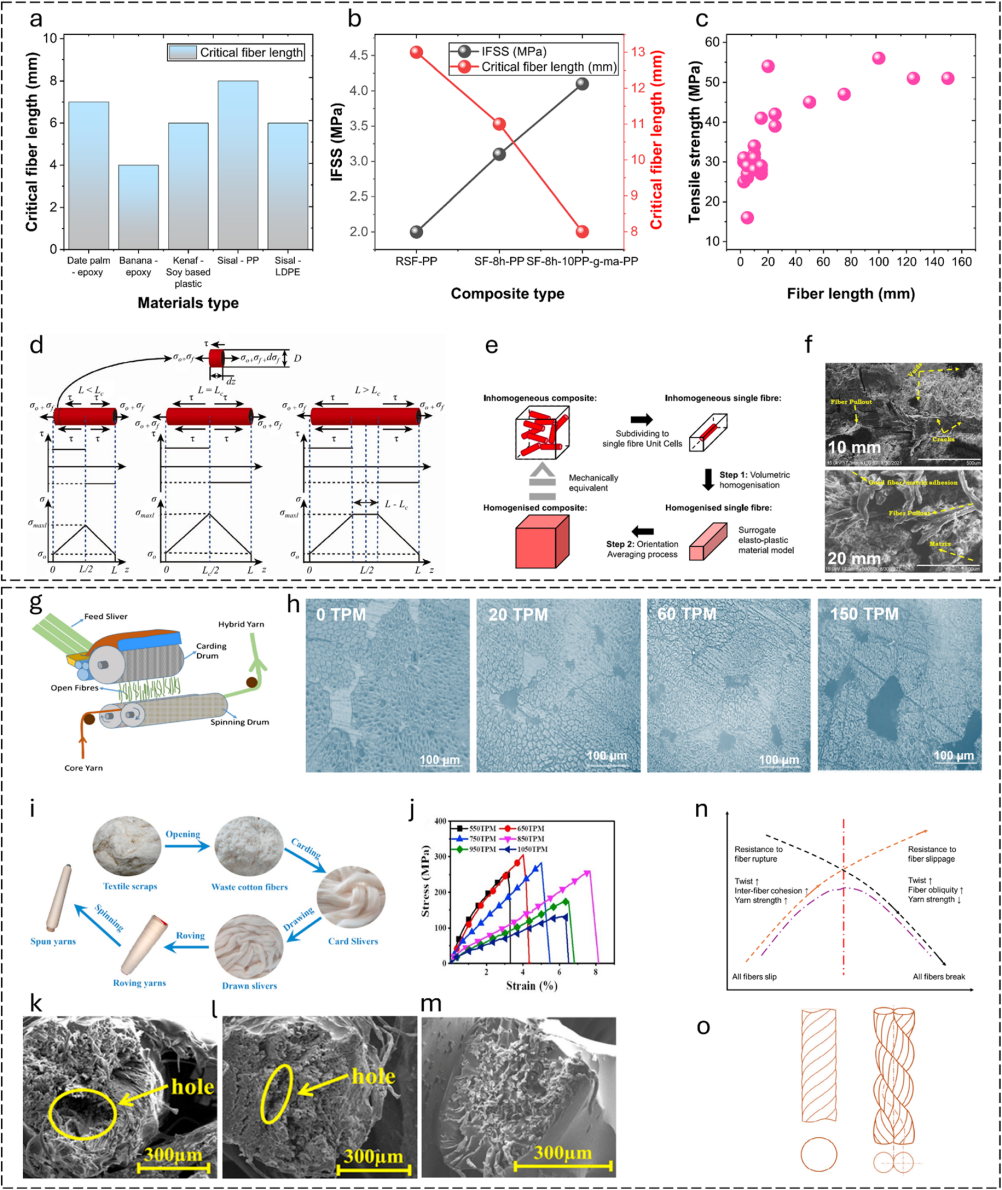
Effect of fiber length and fiber twist on interfacial adhesion and composite properties. (a) Critical fiber length for different composites manufactured using biobased fibers. (b) Interfacial shear strength and critical fiber length of the sisal composites. (a and b) Data adapted from ref. 144, 164 and 176–178. (c) Relationship between tensile strengths of cellulosic fiber-based polymer composites and reinforcing fiber lengths. (d) Schematic of the fiber stress distribution at different fiber lengths. This figure has been reproduced from ref. 170 with unrestricted permission from Elsevier Ltd, licensed Creative Commons CC-BY license, copyright © 2023. (e) Schematic overview of the two-step OA method. This figure has been reproduced from ref. 179 with unrestricted permission from Elsevier Ltd, licensed Creative Commons CC-BY license, copyright © 2023. (f) SEM fractographs of composites manufactured using different fiber lengths. This figure has been reproduced from ref. 168 with unrestricted permission from MDPI, licensed Creative Commons CC-BY license, copyright © 2022. (g) Schematic of the hybrid yarn manufacturing process using DREF-3 spinning. This figure has been reproduced from ref. 171 with permission from Elsevier Ltd, copyright © 2018. (h) Porosity of composites at different twist levels. This figure has been adapted and modified from ref. 172. (i) Flow chart of the yarn preparation process from waste cotton fibers. (j) Stress–strain behavior of single yarn reinforced composites at various twist levels. (k and l) SEM images of the plied yarn-reinforced composite. (m) SEM image of the single yarn-reinforced composite. (i–m) These figures have been reproduced from ref. 173 with permission from Elsevier Ltd, copyright © 2021. (n) Effect of twist on yarn strength. Modified from ref. 174. (o) Geometry and cross-section of single- and two-ply-twisted yarn; left-single twisted yarn, right-two-ply yarn twisted in the opposite direction. Modified from ref. 175.

The minimal fiber length required to enhance the composite properties is called the critical fiber length. Pearson *et al.*^143^ investigated the tensile properties, interfacial adhesion, and microstructure of PBO (poly(phenylene-2,6-benzobisoxazole)) and carbon fiber composites. The interfacial bonding in polymer composites changes with the variation in fiber length relative to fiber length. The critical fiber length required for effective stress transfer in different biofiber composites is shown in Fig. 11a. The modulus, impact strength, and heat deflection temperature (HDT) of kenaf fiber-reinforced soy-based biocomposites improved with increasing fiber length.^164^ Microscopy revealed that the fractured fiber length on the impact fracture surface also increased with fiber length. However, in flax fiber/LDPE composites, the fiber length did not affect the tensile strength. Different fiber lengths (1 and 10 mm) were tested, but the tensile strength remained unchanged regardless of the length.^165^ Bhagat and Ghosh^144^ examined the effect of fiber treatment and addition of polypropylene-grafted maleic anhydride (PP-*g*-MA) on the interfacial shear strength (IFSS) of polypropylene (PP) and sisal fiber (SF) composites. They used the single fiber pull-out test and found that the lowest critical fiber length was 8 mm with the addition of PP-*g*-MA. They concluded that higher fiber–matrix adhesion leads to a lower critical fiber length. Fig. 11b illustrates the interfacial shear strength and critical fiber length of raw and treated sisal/PP composites.

Sreenivasan *et al.*^166^ investigated the effect of fiber length on the dynamic mechanical properties and thermal stability of *Sansevieria cylindrica* fiber reinforced polyester composites by varying the length between 10 and 50 mm. The storage modulus (*E*′) was observed to decrease with increasing fiber length. A lower aspect ratio was attributed to increasing the strength due to fewer fiber defects. However, other factors apart from the fiber length can also be involved in lowering the strength of composites. This could be due to the curling effect of fiber on the composites, and it does not take up the load transfer properly from the matrix along the fiber direction. Composites with shorter fiber lengths (20, 30, and 40 mm) exhibited higher storage modulus values in the glassy region, indicating better reinforcement. However, beyond the glass transition temperature (*T*_g_), the modulus values tended to merge due to softening effects at higher temperatures. A similar trend is seen for loss modulus.^167^ The effect of fiber length (50 and 70 mm) and diameter (200 and 300 µm) on the mechanical, fatigue, and DMA properties of corn husk fiber-reinforced epoxy composites were studied.^167^ A combination of higher fiber length (70 mm) and lower fiber diameter (200 µm) resulted in maximum tensile and flexural strength, while a lower fiber length (50 mm) with a higher fiber diameter (300 µm) resulted in minimum tensile and flexural strength. This could be because, the thicker the fiber, the more flaws it has and *vice versa*. Additionally, a higher fiber length and lower fiber diameter resulted in better DMA characteristics. Anand *et al.*^168^ investigated the effect of fiber length on silane-treated pineapple leaf fiber (PALF) composites. PALF composites were manufactured by varying the fiber length between 5 and 25 mm. The tensile strength and modulus of the PALF composites increased with the increase in the fiber length up to 20 mm. The composites with lower fiber length were considered possibly insufficient to spread uniformly in a polyester matrix, which could result in lesser stress transfer from the matrix to the fibers. Both tensile strength and modulus decreased when the fiber length was further increased from 20 to 25 mm. This results from the resin's inability to adequately infiltrate the spaces between the fibers and resin, leading to suboptimal wetting properties and thus diminishing the effectiveness of stress transmission at the matrix–resin interface. Similarly, the tensile strength and modulus of the bamboo fiber/polystyrene-modified unsaturated polyester composites, when studied for different fiber lengths (2.5–15 mm), increased with the increase in fiber length up to 10 mm (*V*_f_: 0.2–0.3) and decreased when the fiber length was further increased from 10 to 15 mm.^169^

The tensile strengths of cellulosic fibers-based polymer composites studied in previous literature studies for different fiber lengths (5 to 150 mm) were plotted as a function of fiber length in Fig. 11c. The tensile strength of composites increases with the increase in fiber length. This is because the interfacial bonding in composites increases at higher fiber lengths, resulting in an increase in the mechanical properties. Fibers that are too short are unable to hold the fibers together in a preform, resulting in a lack of efficient stress distribution at the interface. However, for a given fiber length, adhesion and composite properties also depend on the preform architecture (*e.g.*, fiber orientation) and preform structure (*e.g.*, areal density and fiber volume fraction). Variation in these parameters affects the permeability, mold filling time, and consolidation behavior of composites.

Fig. 11d illustrates how the fiber length relative to the critical fiber length (*L*_c_) influences the stress transfer and distribution within a fiber–matrix system for oriented fiber composites.^170^ When *L* < *L*_c_, the interfacial shear stress (*τ*) reaches its maximum allowable value before the fiber reaches its tensile strength, suggesting that the interface fails due to debonding rather than fiber fracture. The interfacial shear strength in composites can be determined using the critical fiber length and eqn (3):3
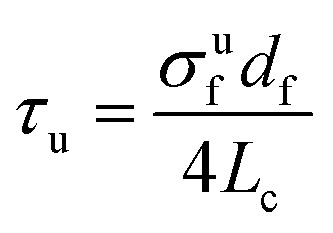
where *σ*^u^_f_ is the fiber fracture strength and *d*_f_ is the fiber diameter. *L*_c_ is the critical fiber length below which fiber debonding will occur and above which fiber fracture will occur.

At *L*_c_, both fiber debonding and failure are possible. At *L* > *L*_c_, the composite fails due to fiber fracture, followed by matrix failure when the axial stress (*σ*_max_) in the fiber reaches the tensile strength of the fibers. Therefore, a short fiber composite typically fails due to fiber pull-out or debonding, whereas a long fiber composite fails due to fiber fracture. Hence, long fiber composites are preferred for structural and semi-structural applications to achieve a stronger fiber–matrix interface and higher composite properties. The average tensile stress *σ*_f_ of a short fiber composite can be determined by integrating *σ*_f_ along the fiber length. The average tensile stress *σ*_f_ can be expressed as eqn (4) and (5) for the case of *L* > *L*_c_ and *L* < *L*_c_, respectively.4
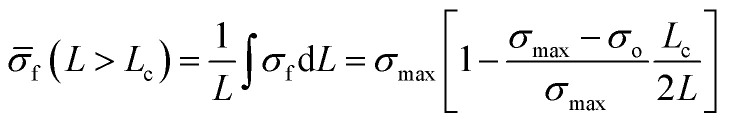
5
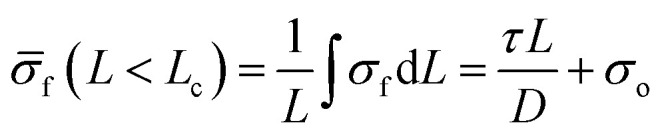


### 4.4 Effect of fiber/yarn twist on the adhesion properties of composites

Bar *et al.*^171^ investigated the effect of hybrid yarn structure on the interfacial adhesion and mechanical properties of flax-PP-based unidirectional composites. The hybrid structured yarn is manufactured using DREF-3 spinning by feeding the PP slivers and then transferring to the yarn forming zone, following wrapping over the core flax yarn (Fig. 11g). The feed rate of the PP silver is varied with respect to a constant core flax yarn feed rate of 40 m min^−1^ to manufacture hybrid yarns of different core-sheath ratios. The flax-PP composites exhibited poor fiber–matrix interfacial bonding at a higher level of core yarn twist (TPI ∼ 8) than those of lower twist (TPI ∼ 0.7), resulting in a decrease in the tensile strength from ∼130 to 118 MPa.

Ma *et al.*^172^ reported that the tensile strength and modulus of sisal yarn-reinforced composites decreased from 212 MPa and 24.3 GPa to 158 MPa and 24 GPa with the incorporation of twist (20 TPM) in sisal yarn. The tensile strength and modulus of composites decreased when the twist level was further increased and exhibited the lowest value at the highest twist level (150 TPM). The decrease in the composite properties with an increase in twist level could possibly be due to the increase in twist angle (maximum at the highest twist level), resulting in the increase in angle between the fiber orientation and the loading direction. The yarn structure tightens as the twist level increases (Fig. 11h), resulting in reduced permeability and incomplete impregnation between the sisal fiber and polymer matrix, lower interfacial bonding and increased voids. This is evident by the maximum composite porosity (3.2%) at the highest twist levels (150 TPM). Thus, the fiber twist in spun yarn reduces composite adhesion.

Yu *et al.*^173^ critically investigated the effect of different twist levels (550–1050 TPM) and twist ratios (0.1–0.5 plied yarn twist to single yarn twist) on the tensile properties of spun yarn-reinforced composites manufactured using waste cotton fibers (Fig. 11i). Fig. 11j shows the stress–strain behavior and tensile strength of the single yarn composites produced at various twist levels (550–1050 TPM). The tensile strength increases when the twist level increases from 550 to 850 TPM, and decreases when the twist level increases beyond this value. This is because the inter-fiber binding force in single yarn is less than that of the component force in the single yarn after reaching the optimum twist. Therefore, the optimum twist level for single yarn is 850 TPM, resulting in a high tensile strength of 172.73 MPa. The decrease in tensile strength when the twist level increased beyond 850 TPM could be due to the misalignment and higher internal stress in the yarn, causing reduced fiber–matrix adhesion in the composite. Similar trends are also observed for plied yarn properties and plied yarn reinforced composites. The tensile strength of plied yarn-reinforced composites manufactured from 6-ply yarn is plotted as a function of varying twist ratios, twist levels, and impregnation rates to understand their effects. The lowest twist level (550 TPM), twist ratio (0.1), and impregnation rate (0.3 cm s^−1^) result in the highest composite properties and *vice versa*. At higher twist levels and ratios, it causes fiber slippage, leading to fiber breakage in fractured composites because of the weak bonding between the epoxy resin and plied yarns. Hence, it is evident that the best interfacial bonding or adhesion is achieved at the optimal twist level and ratios of spun yarn. The SEM images of the fractured surfaces of the plied yarn and single yarn reinforced composites are shown in Fig. 11k–m, respectively. Excessive compactness in plied yarns when compared with single yarns has caused issues during resin impregnation, leading to a porous structure (Fig. 11k) in the fiber–matrix interphase. In contrast, single yarn composites exhibit a non-porous structure characterized by a filled resin-rich region (Fig. 11m), suggesting superior adhesion in composites. This could be due to the loosely bound structure in single yarns, facilitating better impregnation than those of thicker, tighter, and compact structures in plied yarns.

The relationship between the fiber or yarn twist, yarn strength, and the fracture mechanism is shown schematically in Fig. 11n.^174^ At lower twist levels, loosely bound fibers cause fiber slippage and a decrease in yarn strength. The inter-fiber cohesion increases with an increase in twist until it reaches an optimum point and causes an increase in yarn strength due to better fiber integration. Beyond the optimum twist level, the fibers become more oblique to the yarn axis, reducing their ability to bear load efficiently.

Overall, an increase in fiber twist in spun yarn results in a reduction of the fiber–matrix interfacial adhesion. This is attributed to the increased compactness of the yarn and the greater angle formed between the fiber orientation and the loading direction in the twisted yarn, leading to decreased composite adhesion. To address these deficiencies, Zaidi *et al.*^175^ proposed a unique spun yarn configuration by combining two identical twisted yarns in parallel and twisting them in the direction opposite to the initial singles yarns (Fig. 11o). This approach significantly decreases the fiber angle, reducing the misalignment of fibers in the initial twisted single yarn relative to the loading direction, resulting in an increase in composite properties and interfacial adhesion.

### 4.5 Effect of fiber surface roughness on adhesion

**Fig. 12:**
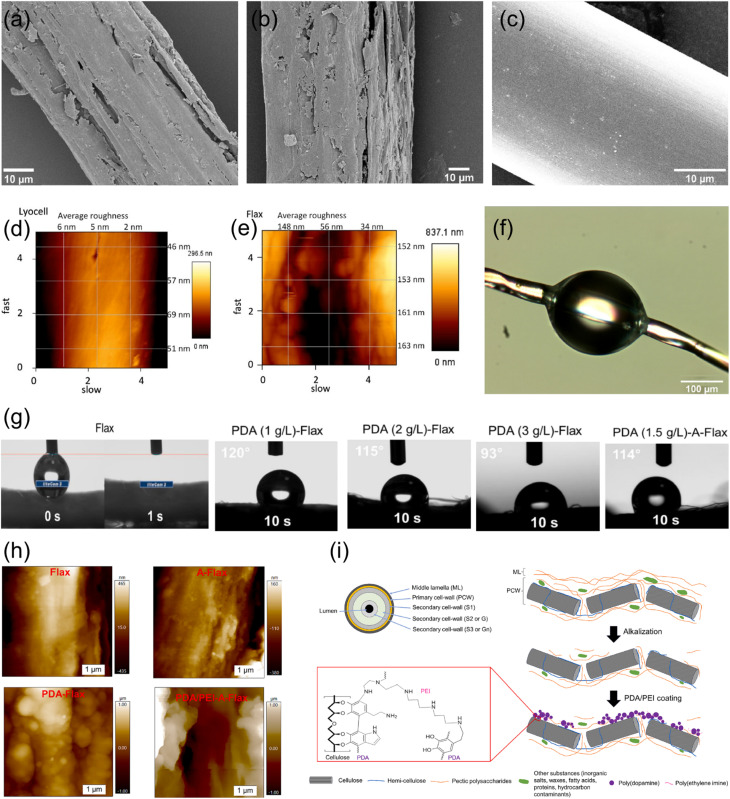
Effect of fiber surface roughness on the adhesion properties of composites. SEM micrographs of (a) kenaf, (b) flax, and (c) lyocell. AFM images of (d) lyocell and (e) flax. (f) Micrograph of a microbond test sample prepared with a lyocell fiber. (a–f) These figures have been reproduced from ref. 129 with permission from Elsevier Ltd, copyright © 2014. (g) Images of the water droplet deposition onto untreated and treated flax. (h) AFM images of Flax, A-Flax, PDA-Flax, and PDA/PEI-A-Flax. (i) Schematic of the arrangement of a single flax fiber and the effect of alkalization and PDA/PEI treatments on the middle lamella and primary cell wall. (g–i) These figures have been reproduced from ref. 180 with permission from Elsevier Ltd, copyright © 2024.

The surface roughness of fibers is typically seen as an important factor affecting adhesion between biofibers and matrices, but the relationship is complex and influenced by multiple factors. For example, in coir/LDPE composites, a smooth fiber with a waxy layer on the surface has resulted in higher interfacial adhesion than a rough surface without waxy substances.^163^ This unexpected outcome indicates that adhesion mechanisms are intricate and not solely dependent on surface roughness. Graupner *et al.*^129^ investigated the correlation between the surface roughness and interfacial adhesion of bast fibers (flax, kenaf) *versus* lyocell fibers. They measured the surface roughness using atomic force microscopy (AFM), finding that bast fibers had a significantly higher average roughness (∼124 nm) compared to lyocell fibers (∼34 nm). This greater roughness resulted in higher interfacial friction, increasing apparent interfacial shear strength (IFSS) values for bast fibers. The effect of fiber surface roughness on the adhesion properties of composites is shown in Fig. 12. The adhesion characteristics of regenerated cellulosic fibers (lyocell) and bast fiber bundles (flax, kenaf) in different polymer matrices, including PLA, PP, and MAPP were examined. It was found that higher interfacial shear strength (IFSS) is measured in PLA and MAPP compared to PP because they are more polar. Bast fibers have better adhesion than lyocell due to rougher surfaces and chemical composition, and pull-out tests yield higher apparent IFSS values than fragmentation tests due to differences in the stress distribution and fiber–matrix friction effects.

Fig. 12a–f provides a comprehensive summary of the impact of the fiber surface roughness on the adhesion behavior of polymer composites, identifying the key structural differences between fibers and treatments. In Fig. 12a–c, SEM micrographs of kenaf, flax, and lyocell fibers exhibit differing surface morphologies, with relatively rough kenaf, moderately rough flax, and smoother lyocell. Such differences in the surface roughness significantly influence the fiber–matrix adhesion since rougher surfaces provide more mechanical interlocking, increasing composite strength. The surface roughness of the lyocell and flax in Fig. 12d and e is further measured by AFM images, which reveal higher roughness values for flax fibers, which may lead to improved wettability and adhesion. Microbond test specimen (Fig. 12f) produced with lyocell fiber visually illustrates the fiber–matrix interaction, giving insight into the adhesion mechanisms at the microscopic level.

Additionally, to achieve the optimum adhesion in flax fiber composites, alkalization and poly(dopamine) (PDA) coating treatment have been tried, with the result of 22.0% enhancement in the interfacial shear strength and 63.2% enhancement in the interlaminar shear strength compared to untreated fibers.^180^ These findings show the importance of fiber surface treatments for improving the mechanical properties and promoting the creation of sustainable natural fiber-reinforced composites. Fig. 12g presents the images of water droplet deposition on untreated and treated flax fibers, illustrating the hydrophobic variations resulting from surface treatments. The untreated flax possesses a greater contact angle, *i.e.*, less wettability, whereas the treated flax fibers exhibit lower contact angles, *i.e.*, improved adhesion potential. AFM images (Fig. 12h) of Flax, A-Flax, PDA-Flax, and PDA/PEI-A-Flax provide a close-up of the surface topography, revealing the effect of alkalization and PDA/PEI treatments on the fiber roughness and structure. The schematic illustration (Fig. 12i) graphically explains the structure of an individual flax fiber and how the middle lamella and primary cell wall are modified by chemical treatments, indicating how the modifications enhance fiber–matrix bonding. This figure emphasizes the significance of the fiber surface roughness and chemical treatments in optimizing the composite adhesion property for improved mechanical performance and durability.

Nano-treatments such as graphene oxide (GO) functionalization, nano-silica coatings, and cellulose nanocrystals (CNCs) have significantly improved the surface roughness, hydrophobicity, and interfacial adhesion. Such nanostructures increase the specific surface area and offer reactive sites that promote mechanical interlocking and chemical bonding with matrices such as PLA.^181^ In addition, barrier properties and thermal stability were determined to be enhanced by nanoclay treatments, as well as composite durability.^182^ In addition to traditional roughness evaluation, newer techniques, such as machine learning workflow with unsupervised learning^183^ and colorimetric roughness measurements using spectrophotometers,^184^ have been applied in recent years to assess the surface roughness of polymer-based composites.

## 5. Effect of surface treatments on the biofiber microstructure and adhesion

The surface treatment is employed on fibers to tailor the wettability, enhance the surface chemical activity, and increase the surface roughness, resulting in enhanced chemical bonding and mechanical interlocking between the fibers and polymer matrix. In order to maximize the durability and performance of polymer matrix composites, an optimal stress distribution from the polymer matrix to the reinforcing fiber is required. However, the interfacial bonding strength of the raw reinforcing fibers, which did not undergo any surface treatment, is relatively low due to its chemical inertness and poor wettability, causing fiber debonding or fiber pull out when subjected to loading conditions. Surface treatment can be applied to fibers in the form of chemical or physical modifications.

### 5.1 Chemical treatments

**Fig. 13:**
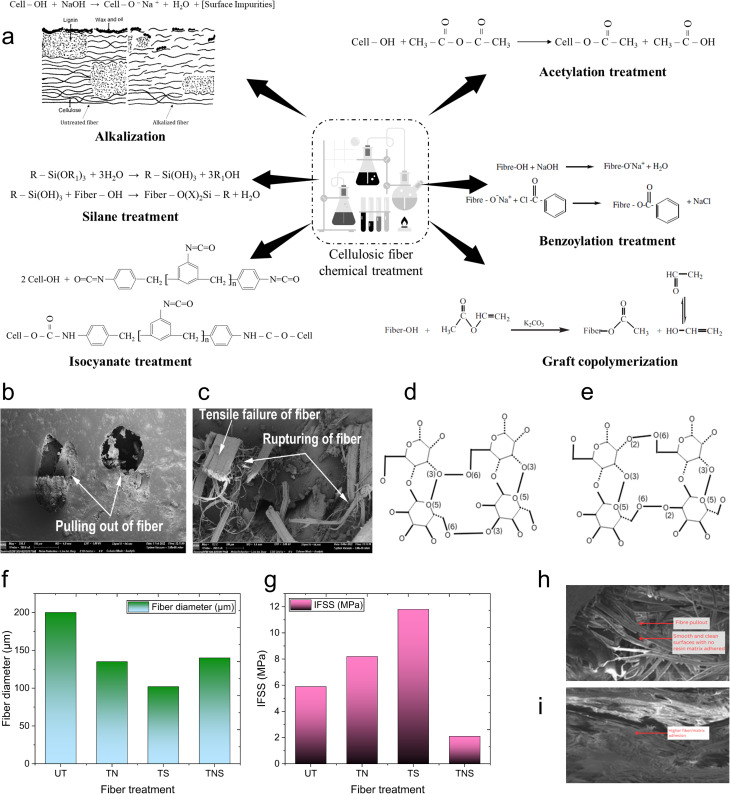
Effect of surface treatments on composite adhesion. (a) Mechanism and interaction of the reagent functional groups of different surface treatments with cellulosic fiber structures. This figure has been reproduced from ref. 57 with permission from Elsevier B.V., copyright © 2023. (b and c) SEM photographs of the interface of (b) untreated and (c) alkali-treated cellulosic fiber reinforced biocomposites. (b and c) These figures have been reproduced from ref. 193 with permission from John Wiley and Sons, copyright © 2023 Society of Plastics Engineers. (d and e) Hydrogen bonding pattern for (d) cellulose I and (e) cellulose II. (d and e) This figure has been reproduced from ref. 124 with permission from Elsevier B.V., copyright © 2010. (f) Diameter of SPF before and after the treatments. (g) IFSS of SPF before and after the treatments. (f and g) Data adapted from ref. 137. (h and i) SEM micrographs of (h) untreated jute fiber/HDPE, (i) plasma-treated jute/HDPE at 90 W in RF. (h and i) These figures have been reproduced from ref. 194 with permission from Elsevier B.V., copyright © 2011.

Natural fibers have been treated with chemicals like alkali, silane, acetic anhydride, isocyanates, benzoylation, and peroxides to increase their compatibility with hydrophobic polymer matrices.^108,114,185^ These treatments utilize reagent functional groups that react with the fiber structures shown in Fig. 13a, altering their composition and enhancing the interaction between the cellulose surface and the matrix, thereby ensuring good compatibility.^16^ Acetyl treatment, also known as esterification, reduces the hydrophilicity of natural fibers by reacting acetyl groups (CH_3_CO–) with hydroxyl groups (–OH) in the amorphous region, thereby removing moisture.^186^ It also creates a polymer coating from maleic anhydride, further decreasing hydrophilicity.^187^

Alkaline treatment is the most used chemical treatment for plant fiber composites due to its cost-effectiveness.^15,188,189^ The treatment process removes portions of hemicelluloses, lignin, pectin, wax, and oil-covering materials, typically resulting in a clean but rough surface that enhances interfacial adhesion.^139,140^ However, when hemp fiber was treated with 8% NaOH, the removal of hemicellulose and lignin led to a smooth fiber surface.^190^ Similarly, treating banana fiber with 1% NaOH also resulted in a smooth surface, demonstrating that the removal of these components can sometimes lead to a reduction in surface roughness.^191^ Mildly alkaline sodium bicarbonate treatment also reduces hemicellulose and lignin.^192^ Alkaline treatments eliminate micro voids, resulting in a more uniform fiber surface, strengthening the stress transfer capacity between the cells. Additionally, this process reduces the fiber diameter and increases the aspect ratio, leading to improved interfacial adhesion between the fiber and the matrix. Fig. 13b and c show the SEM photographs of the interface of untreated and sodium hydroxide-treated cellulosic fiber-reinforced biocomposites, respectively, illustrating the compatibility between fiber and the matrix.^193^ The untreated sample shows weak adhesion at the interface, whereas the treated sample demonstrates enhanced interfacial bonding, indicating improved compatibility with the matrix.

Crystalline cellulose, crucial for fiber–matrix adhesion, does not easily bond with new chemicals due to its crystalline regions composed of tightly bonded hydroxyl groups, making them difficult to access and penetrate.^160,195^ Alkaline treatment alters the structural alignment of highly crystalline cellulose, creating amorphous regions by causing the fiber cell wall to swell.^19,147^ The process of alkylation is industrially known as mercerization. Cellulosic fibers are essentially more receptive to dyes and chemicals after mercerization. The mechanism involves the conversion of elementary fibrils into another crystal form, known as cellulose II.^196^ This conversion of structure occurs within single nanocrystals by disrupting intrachain H-bonding in anhydrous cellulose I when it interacts with Na^+^ ions, allowing adequate water to penetrate and swell the fibrils, and permitting the cellulose chains to create the space required for folding back.^196^ The fundamental differences between cellulose I and II are their chain orientations and hydrogen bonding patterns: cellulose I has parallel chains with O6–H⋯O3 hydrogen bonding, while cellulose II has antiparallel chains with O6–H⋯O2 bonding.^197^Fig. 13d and e represents the hydrogen bonding pattern for cellulose I (A) and cellulose II (B). This process partially removes hydrophilic hydroxyl groups, improving moisture resistance.^198^

Overall, the alkali-treated cellulosic fibers exhibited higher crystallinity index.^199^ Wang *et al.*^200^ observed that IFSS increased in composite samples treated with 1%, 4%, and 7% NaOH compared to untreated samples. However, IFSS decreased in samples treated with 7% NaOH compared to those treated with 4% NaOH, likely due to cellulose crystal damage and increased polarity at higher alkali concentrations. High-concentration NaOH alkalization, followed by acetylation, effectively removes hemicellulose and lignin, while silane treatment forms couplings with fiber constituents without removing these components.^19,147,190^ Liu *et al.*^201^ treated corn stalk fiber with silane coupling agents (aminopropyltriethoxysilane) in concentrations of 1%, 5%, 9%, and 13%. The treatment enhanced interfacial adhesion by creating a cleaner and slightly rougher surface, thus improving mechanical interlocking. The improvement in the interfacial shear strength (IFSS) of various fiber–resin composites achieved through different chemical treatments, highlighting the effectiveness of each treatment in enhancing fiber–matrix bonding, is presented in Table 4.

**Table 4:** Improvement in the interfacial shear strength (IFSS) of various composites upon different chemical treatments^a^

Types of treatment	Fiber	Resin	% IFSS improvement	Method used	Reference
Alkali (4% NaOH)	Jute	PLA	67.55	SFPT*	202
Alkali treatment followed by silane (NaOH + silane)	Jute	PLA	112.3	SFPT*	202
0.02% w/v Potassium permanganate aqueous solution	Jute	PP	25	YPT*	203
0.02% w/v Potassium dichromate aqueous solution	Jute	PP	61	YPT*	203
0.02% w/v Sodium perborate trihydrate aqueous solution	Jute	PP	71	YPT*	203
Alkali (5% NaOH)	Hemp	PLA	105.68	SFPT*	204
0.5 wt% Silane coupling agent [3-(2-aminoethylamino) propyl trimethoxy silane] in acetone	Hemp	PLA	48.11	SFPT*	204
Alkali treatment followed by saline (NaOH + silane)	Hemp	PLA	77.7	SFPT*	204
5 wt% Maleic anhydride in acetone	Hemp	PLA	−3.96	SFPT*	204
Acetylation (acetic anhydride)	Hemp	PLA	13.33	SFPT*	204
Alkali (6% NaOH)	Sisal	PP	173	SFPT*	205
Acetylation (ethyl acetate containing H_2_SO_4_ as a catalyst)	Sisal	PP	435	SFPT*	205
Alkali (2% NaOH at room temperature 12 h followed by 7.5% NaOH for 90 min)	Sisal	PLA	150	PT*	206
2% v/v Saline	Sisal	PLA	120.83	PT*	206
Alkali treatment followed by saline (NaOH + silane)	Sisal	PLA	141.67	PT*	206
Alkali (4% NaOH)	Flax	PP	8.36	PT*	207
10% MAPP	Flax	PP	15.06	PT*	207
Alkali (20% NaOH)	Flax	PP	8.42	PT*	207
10% MA	Flax	PP	−22.01	PT*	207
10% MAPP	Flax	PP	15.07	PT*	207
2.5% Vinyl trimethoxy silane	Flax	PP	3.09	PT*	207
Alkali (6% NaOH)	SPF	P	59	DT*	137
2% Saline	SPF	P	115	DT*	137
Benzoylation using benzoyl chloride (C_7_H_5_ClO)	SPF	Epoxy	143.2	MDT*	208
Alkali (5% NaOH)	Cattail	Epoxy	86.83	MBT*	199
Alkali (4% NaOH)	Bamboo	Epoxy	100.3	MBT*	200

a *SFPT-Single fiber pullout test. *YPT-Yarn pullout test. *PT-Pull out test. *MDT-Microdroplet test. *MBT-Micro bond test.

Alkali treatments, particularly with NaOH, always enhance fiber–matrix adhesion, with sisal fibers showing a tremendous 173% enhancement in PP composites.^205^ Double treatments of alkali–silane provide additional enhanced IFSS enhancement, with jute fiber/PLA composites exhibiting 112.3% improvement,^202^ demonstrating the synergistic influence of the hydroxyl group removal and silane coupling reaction. Acetylation treatment used on sisal/PP composites (435%), significantly improves adhesion by surface polarity adjustment.^205^ Moreover, oxidizing agent treatments by potassium permanganate and sodium perborate increase jute/PP composites' IFSS between 25% and 71%.^203^ Benzoylation treatments, when applied to SPF/epoxy composites, show an improvement of 143.2%, thereby establishing their role to enhance fiber–resin interaction.^208^ Maleic anhydride treatment surprisingly shows an adverse IFSS effect of −3.96%, indicating probable incompatibility.^204^ These findings underscore the significance of chemical surface treatments for optimizing adhesion characteristics to ensure long-term, high-performance biofiber composites in structural and engineering functions.

Zaman and Khan^142^ assessed the interface quality and interfacial shear strength (IFSS) of untreated, NaOH-treated, silane-treated, and NaOH-silane-treated PALF-epoxy composites using a single-fiber fragmentation test. They found that treated samples showed significantly increased IFSS, indicating improved adhesion, with silane-treated fibers exhibiting the highest IFSS and the smallest diameter of 41.2 µm. Similarly, the effects of alkaline and silane treatments on the physical, chemical, mechanical, and morphological characteristics of sisal plant fibers (SPF) were investigated.^137^ Treated SPF fibers exhibited smaller diameters compared to untreated fibers. Silane-treated fibers had the smallest diameter and showed the highest IFSS, approximately 115% higher than untreated fibers. Alkaline-treated fibers also demonstrated improved IFSS, with a 59% increase over untreated fibers. The reduction in fiber diameter due to surface treatments, especially with silane, contributed to better mechanical performance. Fibers with smaller diameters offer a higher surface area-to-weight ratio, ensuring better dispersion and proper wetting, which creates a strong interface and enhances the load-transfer mechanism.^15^Fig. 13f illustrates the diameter of sugar palm fiber (SPF) under different treatments: untreated (UT), NaOH treated (TN), saline treated (ST), and NaOH/saline treated (TNS), while Fig. 13g illustrates the IFSS of sugar palm fiber (SPF) under different treatments: untreated (UT), NaOH treated (TN), saline treated (ST), and NaOH/saline treated (TNS).

**Fig. 14:**
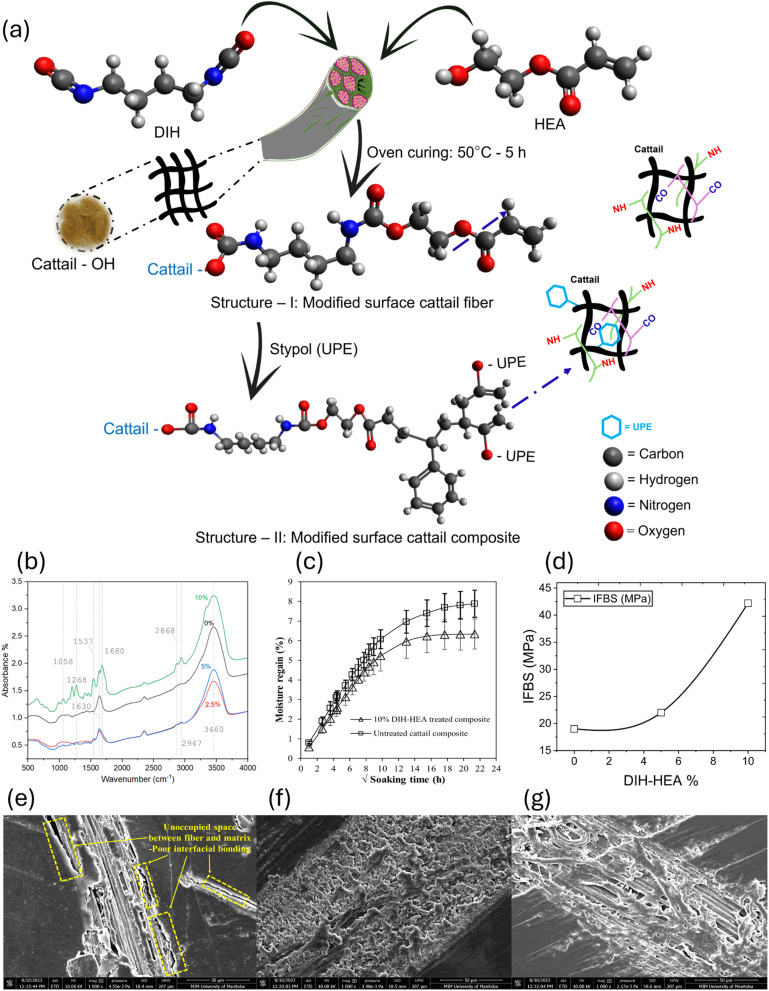
Chemistry and mechanism of the surface treatment of cattail fiber-reinforced composites. (a) Surface treatment chemistry. (b) FTIR spectra. (c) Moisture absorption. (d) Interfacial bonding strength of treated and untreated composites. (a and d) Data adapted from ref. 71. (b and c) These figures have been reproduced from ref. 71 with unrestricted permission from Wiley Periodicals LLC, licensed under CC-BY-NC-ND license, copyright © 2023. (e–g) SEM images of surfaces of treated and untreated cattail fiber composites (e) untreated; (f and g) treated.

Cattail fibers were subjected to isocyanate treatment using 1,6-diisocyanatohexane (DIH) and 2-hydroxyethyl acrylate (HEA) for manufacturing polymer composites. The mechanism and chemistry of manufacturing modified surface cattail composites are illustrated in Fig. 14a.^71^ The covalent bonding between the treated fibers and DIH-HEA molecules was confirmed by the ATR-FTIR spectra (Fig. 14b) peaks at 1537 cm^−1^ and 1268 cm^−1^, associated with the vibration of the N–H group and C–N group, respectively, confirming carbamate linkage in treated cattail fibers. The equilibrium moisture absorption (*M*_m_) of the treated cattail composites (Fig. 14c) decreased from 7.88 (±0.69)% to 6.34 (±0.76)% because of the incorporation of these functional groups onto the fibers. These consequently resulted in ∼122% increase in interfacial bonding strength in composites after surface treatment (Fig. 14d), suggesting the enhancement in fiber–matrix bonding in treated composites (Fig. 14f and g) when compared with untreated cattail composites (Fig. 14e).

### 5.2 Physical treatments

Physical methods such as plasma treatment, electric radiation, ultraviolet (UV) exposure, and corona treatment can alter fibers' structural and surface properties, breaking down fiber bundles into individual filaments.^209^ Atmospheric air plasma treatment (AAPP) increases fiber surface roughness by creating numerous micro-holes, transforming smooth surfaces into porous structures.^210^ Baltazar-Y-Jimenez *et al.*^211^ found that AAPP treatment affects lignocellulosic fibers' mechanical properties and interfacial behavior. IFSS increased slightly for flax, hemp, and sisal fibers after 1 minute, but decreased with extended treatment for abaca and sisal due to weak boundary layer formation. Plasma etching and high temperatures after 1 minute of AAPP treatment create a rough surface with cracks, pits, and corrugations, possibly enlarging pre-existing flaws. After 3 minutes, SEM analysis shows more severe deterioration with increased and deeper cracks, pits, and corrugations. Physical treatments improve mechanical properties and enhance interfacial adhesion without affecting the fiber's chemical composition.^209^ The increase in IFSS following plasma treatment is attributed to a combination of factors, which leads to alterations in surface chemistry by introducing functional groups. For atmospheric air gas plasma treatment, these groups are likely carboxyl, peroxy, hydroperoxy, amine, and nitroso groups.^211^Fig. 13h shows the SEM of untreated jute fiber/HDPE composites, demonstrating the fiber–matrix interface with potential weak bonding. In contrast, Fig. 13i shows the SEM of plasma-treated composites, illustrating improved fiber–matrix adhesion and surface characteristics after treatment, highlighting better interfacial bonding. Choi *et al.*^212^ investigated henequen fiber-reinforced polypropylene biocomposites treated with electron beam irradiation. They assessed the interlaminar shear strength (ILSS) to evaluate the interfacial adhesion between the fibers and the polypropylene matrix. AFM images revealed that the irradiation removed pectin, waxy materials, and impurities from the fiber surfaces, altering their morphology. This removal resulted in the formation of small pores (1–0.01 µm), increasing the total surface area and porosity, which enhanced the fiber-polymer adhesion as confirmed by ILSS tests.

Tables 5 and 6 provide a synopsis of the plasma treatment processes and structural changes enhancing the interfacial shear strength (IFSS) respectively, in biofiber-polymer composite materials. Low-pressure air plasma treatment, dielectric barrier discharge, atmospheric pressure glow discharge, and liquid plasma processing successfully enhance the fiber–matrix adhesion with increases ranging from 20% to 331.1% based on the fiber type and conditions employed for the plasma. Non-thermal plasma treatment of hemp/epoxy composites achieved a 331.1% increase in IFSS, validating the effectiveness of O_2_ and air-plasma treatments.^217^ Liquid plasma treatments using NaHCO_3_ media also enhanced the coir/epoxy composites, increasing IFSS by 72–79%,^119^ which clearly shows the working of alkaline-based plasma treatments in surface-modifying properties. Similarly, air pressure glow discharge treatment of flax/HDPE and flax/PE composites improved IFSS by 40–45%, justifying air and argon plasma interaction for modifying fiber chemistry.^215^ In addition, structural modifications play a crucial role in enhancing the adhesion performance in biofiber composites. Hydrophobicity of the fibers is enhanced through the degradation of amorphous hemicellulose and lignin, increasing the matrix compatibility.^199,219^ Thinning the diameter of the fibers enhances stress distribution and interfacial shear strength.^142,222^ Improved surface roughness increases mechanical interlocking, and increased crystallinity values and micropores increase fiber stability and interlaminar shear properties.^212^ These findings emphasize the importance of surface and plasma treatment in optimizing durability in cellulosic fiber composites for engineering applications.

**Table 5:** Improvement in the interfacial shear strength (IFSS) of various fiber–resin composites upon different types of physical treatment^a^

Types of treatment	Composite type	Pressure	Temp (°C)	Plasma gas	Power (W)	Time (seconds)	IFSSo (MPa)	IFSSt (MPa)	% IFSS improvement	Method used	Reference
Low-pressure air plasma treatment	Sisal/pp	2 torr	25–200	Air and Ar	30	30–120	2.5	3.2 (Ar), 2.7 (Air)	28 (Ar), 24 (Air)	Pull out test	213
Dielectric barrier discharge (1 atm)	Henequen/HDPE	1 atm	25	Ethylene	NA	60–480	2.4	3.4	41.67	Pull out test	214
Liquid plasma treatment (water medium)	Coir/epoxy	20 kPa	25	Microwave plasma	600	180–240	2.82	4.01–4.74	42–68	Pull out test	119
Liquid plasma treatment (8% NaHCO_3_ medium)	Coir/epoxy	20 kPa	25	Microwave plasma	600	180–300	2.82	4.23–4.58	50–62	Pull out test	119
Liquid plasma treatment (10% NaHCO_3_ medium)	Coir/epoxy	20 kPa	25	Microwave plasma	600	180–300	2.82	4.65–4.75	65–68	Pull out test	119
Liquid plasma treatment (12% NaHCO_3_ medium)	Coir/epoxy	20 kPa	25	Microwave plasma	600	180–300	2.82	4.84–5.04	72–79	Pull out test	119
Atmospheric pressure glow discharge	Flax/HDPE	N/A	N/A	Air and Ar	100–300	120	5.5	7.7	40	Pull out test	215
Atmospheric pressure glow discharge	Flax/PE	N/A	N/A	Air and Ar	100–300	120	5.5	7.975	45	Pull out test	215
Atmospheric pressure plasma jet	Ramie/PP	1 atm	20	He (alcohol pre-soaked)	40	8–24	16.1	19.2–23.5	20–46	Microbond test	216
Non-thermal plasma treatment	Hemp/epoxy	1 atm		Air or O_2_	15/25	120–600	13.5	44.7	331.1	Fragmentation test	217
Plasma treatment	Jute/PLA	N/A	N/A	Helium and acrylic acid	3 kV	30–120	3.59	6.84	90	Microbond test	218

a IFSSo = IFSS before treatment; IFSSt = IFSS after treatment.

**Table 6:** Effects of surface treatment-induced structural changes on fiber adhesion

Structural change	Fiber^a^	Effect on adhesion	Reason/evidence	Method	Reference
Removal of amorphous hemicellulose, lignin	T, RH, CW, A, B	+ve^b^	Increased hydrophobicity due to reduced polar group	FTIR analysis	199 and 219–221
Reduced diameter	Ca, PF SPF	+ve	Interfacial shear strength	SEM scanning electron microscope (for diameter); SFMT, SFPT, DT	137, 142 and 222
Increased surface roughness	Ca, RH, A	+ve	Increased interfacial shear strength	SEM, AFM, SFPT	129, 199 and 219
Increased crystallinity index	S, Ca, RH	+ve	—	XRD analysis	199, 205 and 219
Increased surface area and micropores	H	+ve	Increased inter laminar shear strength	Mercury porosimetry using Autopore IV 9500; SBS test according to ATM D-2344	210 and 212

a J: Jute, K = kenaf, Ca = Cattail, B = Banana, R = Rice husk, H = Henequen, CW = Cotton Waste, A = Abaca, PF = Pineapple leaf fiber, SPF = Sugar Palm fiber, SBS test = Short beam shear.

b +ve: Positive.

## 6. Future directions and outlook

Increased advancements in the adhesion chemistry of cellulosic fibers and polymer matrices hold tremendous potential for improved composite performance, durability, and sustainability. The future study should consider synthesizing bio-based coupling agents that evolve greater fiber–matrix interaction without recourse to traditional synthetic treatments. Enzymatic surface modifications are an alternative possibility that has the potential to selectively modify composition without affecting structure. In addition, surface modification through nanotechnology, such as graphene oxide functionalization and nano-silica coating, could significantly enhance fiber hydrophobicity and interfacial adhesion. These approaches provide a green way to achieve optimum adhesion chemistry with zero environmental cost.

Another potential research direction involves the application of machine learning approaches to predict and optimize fiber adhesion properties as a function of structural modification. Computational modeling, including molecular dynamics modeling and artificial intelligence-based adhesion prediction software, can be utilized to identify optimal fiber treatments and polymer compatibility before experimental validation. This strategy will minimize the loss of materials, maximize research efficiency, and speed up the pace of next-generation fiber–matrix composites. Moreover, exploring biofiber blends using hybrid reinforcement approaches, merging natural fibers with bio-polymer matrices, may increase the scope of applications in biodegradable and recyclable composite materials.

Mass-scale application of advanced surface engineering technologies remains imperative to commercializing high-performance composites of biofibers. Researchers must divert focus to cost-effective plasma processing, silane-based adhesion promoters, and green chemical treatments that can be applied in mass production. Moreover, long-term environmental stability testing under changing conditions such as moisture, UV, and mechanical loading will be given due attention in upcoming research to determine the adhesion stability for various applications. Collaborative research with industry and practitioners for sustainability will optimize the fiber–polymer interfaces, and the biofiber composites will become increasingly more relevant in car, aerospace, and structural engineering applications.

Polymer resin adhesion to biofibers is a three-dimensional interaction of the morphology of the fiber, chemical structure, and surface treatment, all of which directly impact the strength of interfacial bonding in composites. Fiber diameter, roughness of the surface, hemicellulose, and lignin are controlling parameters that significantly impact adhesion effectiveness. While they are natural components of the biofiber, hemicellulose and lignin add weak boundary layers, interfere with the matrix–fiber bonding, and impact mechanical integrity. Their hydrophilic nature leads to water absorption that degrades the adhesion stability and durability in polymer composites. Thus, these components must be removed or converted to improve compatibility with hydrophobic matrices.

Maximizing fiber morphology and surface modification technology is critical to developing high-performance biofiber composites with improved mechanical strength and environmental durability. With the inclusion of advanced adhesion promotion technologies, the future of bio-based sustainable composite materials will emerge, taking innovations in structural engineering, aerospace, automobile, and biomedical technology to new heights. Advanced adhesion chemistry will enable researchers to shatter the frontiers of bio-composite engineering, offering green and high-strength material technologies.

We must focus on multidisciplinary collaborations that blend materials science, environmental engineering, and computational modeling to implement these advances effectively. The development of normative adhesion testing methods, such as interfacial shear strength (IFSS) tests under service conditions, will provide uniform benchmarks for composite performance. Encouraging industry-university collaboration for large-scale testing and certification will make the uptake of optimized biofiber composites in commercial markets a reality. These advancements would assist in accomplishing the development of long-term, sustainable, and high-performance cellulosic fiber–polymer composites capable of supporting green material engineering advancements.

## Conflicts of interest

The authors declare that they have no competing financial or non-financial interests.

## Data Availability

No new datasets were generated during the current study. All data cited in this review are available in the referenced publications.
